# Protective effects of resveratrol on the ethanol‐induced disruption of retinogenesis in pluripotent stem cell‐derived organoids

**DOI:** 10.1002/2211-5463.13601

**Published:** 2023-03-30

**Authors:** Hongyu Li, Lixiong Gao, Zi Ye, Jinlin Du, Wen Li, Liqing Liang, Quan Zeng, Jiafei Xi, Wen Yue, Zhaohui Li

**Affiliations:** ^1^ Medical School of Chinese PLA Beijing China; ^2^ Senior Department of Ophthalmology The Third Medical Center of Chinese PLA General Hospital Beijing China; ^3^ Stem Cell and Regenerative Medicine Lab Beijing Institute of Radiation Medicine China; ^4^ South China Research Center for Stem Cell and Regenerative Medicine Souty China Institute of Biomedicine Guangzhou China

**Keywords:** ethanol, fetal alcohol syndrome, PI3K‐AKT signalling, resveratrol, retinal organoids

## Abstract

Prenatal alcohol exposure‐induced fetal alcohol syndrome (FAS) can lead to serious maldevelopment in many organ systems, including the eyes. In the present study, the effects of alcohol exposure on early development of the human retina and the therapeutic effects of resveratrol on alcohol‐induced neural retinal damage were observed for the first time in an *in vitro* retinal organoid model. We report that the number of proliferating and apoptotic cells decreased and increased, respectively, following ethanol treatment. In addition, the number of PAX6^+^ cells and migrating TUJ1^+^ cells decreased after ethanol exposure. However, pretreatment with resveratrol prevented all of these negative effects. Using RNA sequencing and immunofluorescence, we identified activation of the PI3K‐AKT signalling pathway as the possible mechanism through which resveratrol protects the retina from alcohol‐induced damage. These results suggest that while ethanol exposure can restrict the growth of the human retina and impede the development of specific retinal cells, pretreatment with resveratrol may be a feasible method for preventing these effects.

## Graphical abstract

Pluripotent stem cell‐derived organoids *in vitro* can be widely used in disease modelling and drug screening. Based on a retinal organoid model, we found that alcohol significantly inhibited retinal cell proliferation and promoted cell apoptosis. Furthermore, pretreatment with resveratrol can reduce alcohol‐induced retinal cell damage by activating the PI3K‐AKT signalling pathway.

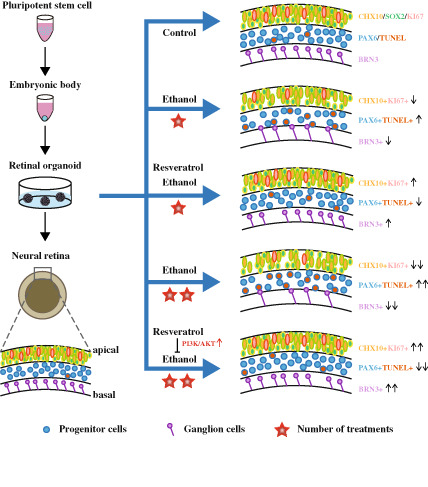

**Abbreviations**
ANOVA: analyses of variance
AP: alkaline phosphatase
BAC: blood alcohol concentration
BMP4: recombinant human bone morphogenetic protein 4
CPM: counts per million
DAPI: 4′; 6‐diamidino‐2‐phenylindole
DEG: differentially expressed gene
EB: embryoid body
EDTA: ethylene diamine tetraacetic acid
ESC: embryonic stem cell
EtOH: ethanol
FAS: fetal alcohol syndrome
FBS: fetal bovine serum
GFAP: glial fibrillary acidic protein
gfCDM: growth factor‐free chemically defined medium
GO: gene ontology
GSEA: gene set enrichment analysis
KEGG: Kyoto Encyclopedia of Genes and Genomes
KSR: knockout serum replacement
NR: neural retina
NRDM: neural retina differentiation medium
PBS: phosphate‐buffered saline
PFA: paraformaldehyde
PSC: pluripotent stem cells
RGC: retinal ganglion cell
RO: retinal organoid
RSV: resveratrol
RT‐qPCR: real‐time quantitative polymerase chain reaction
TUNEL: terminal deoxynucleotidyl transferase‐mediated dUTP nick end labelling

Fetal alcohol syndrome (FAS) is a neurodevelopmental disorder caused by prenatal alcohol exposure [1]. The global prevalence of FAS is 0.77%, with that being higher (2–5%) in Europe and North America [2]. It has been reported that one out of 33 pregnant women in the United States has a history of binge drinking (blood alcohol concentration to 80 mg·dL^−1^ or above) [3, 4, 5]. Unrelenting alcohol consumption during pregnancy and the resultant increase in fetal exposure to alcohol contribute to a higher likelihood of FAS development, which is always accompanied by severe cognitive and behavioural abnormalities in early childhood [6]. In the USA, approximately $5.4 billion per year is spent on treating FAS [7], placing a heavy burden on both society and family.

Eye defects, including microphthalmia, optic nerve hypoplasia, cataracts and low visual acuity, are frequently observed in children with FAS [8, 9]. A study based on monkeys found that the retina aged more quickly and the expression of glial fibrillary acidic protein (GFAP) was increased in the retinal ganglion cell (RGC) layer following ethanol (EtOH) exposure compared with the corresponding parameters in the control. This was indicative of strong astrogliosis in the monkey retina [10, 11]. A recent study confirmed that the retinal nerve fibre layer is thinner in young adults with FAS [12]. These results imply that RGCs may be the most vulnerable cell types during FAS pathogenesis.

Although no specific and effective drugs are currently available for the prevention or treatment of FAS, several antioxidants, such as vitamin C, folic acid, astaxanthin, choline and resveratrol (RSV), have recently emerged as possibly protective agents in alcohol‐exposed foetuses based on animal and cellular evidence [6, 13, 14, 15, 16]. Among them, RSV, a phytoalexin and the polyphenolic compound found in plants, has been found to play multiple protective roles, including in antioxidation, anti‐inflammation, cardiovascular protection and antiageing [17, 18, 19, 20, 21]. Our previous work showed that RSV pretreatment protects against EtOH‐induced defects in hippocampal neurogenesis in postnatal mice. Recent studies have confirmed that RSV can attenuate retinal inflammation and neural apoptosis in diabetic rats [22, 23]. More specifically, RSV mitigates RGCs loss induced by retinal ischemic injury [24]. However, whether RSV plays a protective role in EtOH‐induced retinal injury during embryonic development remains unknown. Unravelling the detailed mechanism of EtOH exposure‐related human retinal injury, which has not yet been fully elucidated, is important for developing effective management methods.

Unfortunately, ethical barriers hinder the direct collection of evidence from humans. Animal models and *in vitro* cell experiments have played major roles in elucidating the pathogenesis of FAS [25, 26]. However, differences between animals and humans, lack of histologic structure in conventional cell models and the inability to observe long‐term effects have prevented the determination of clear results. Consequently, researchers have now turned to a newly generated stem cell model, the organoid, to overcome these barriers. Based on human pluripotent stem cells (PSCs), organoid models can mimic both structurally and functionally the development of specific organs, including the brain, retina, intestine and kidneys [27, 28, 29, 30]. More importantly, their development‐mimicking characteristics make organoids an accurate and promising model for disease pathogenesis, drug discovery and regenerative medicine [31]. In the present study, using modified SFEBq culture and EtOH treatment, we observed embryonic body growth, cell proliferation and apoptosis, and subcellular development within the neural retina. We also explored the protective effects of RSV on EtOH‐induced disruption of retinogenesis. Using RNA sequencing, we analysed the gene‐expression alterations following EtOH treatment and RSV pretreatment and screened out potential signalling pathways.

## Materials and methods

### Culturing of human embryonic stem cells (hESCs)

WA09 hESCs (H9‐ESCs) were purchased from the Wicell Research Institute and maintained using a feeder‐free culture protocol. Briefly, hESCs were first stabilised in germ‐free 6‐well plates (Thermo, Waitham, MA, USA) coated with 1% Matrigel (Corning, Teterboro, NJ, USA). Then, culture medium was changed daily using the mTeSR Plus complete kit (STEMCELL Technologies, Vancouver, Canada). For passaging, hESCs monolayers were digested into small masses with 0.5 mM ethylene diamine tetraacetic acid (EDTA, PH = 8.0, Biosharp, Hefei, Anhui, China) every 4 days [32]. All experiments involving human cells were conducted in accordance with the Tenets of the Declaration of Helsinki and approved by the ethics committee of the General Hospital of the People's Liberation Army.

### Immunofluorescence examination of H9‐ESCs

H9‐ESCs seeded on the 1% Matrigel‐coated round coverslip in 24‐well plate on day 4 were fixed with 4% paraformaldehyde (PFA, Servicebio, Wuhan, Hubei, China) for 30 min at room temperature. After treating with 0.2% Triton X‐100 and 10% donkey serum and incubating at room temperature for 30 min, cells were incubated with the following primary antibodies in 10% donkey serum overnight at 4 °C: rabbit anti‐NANOG (1/200, Abcam, UK), rabbit anti‐OCT4 (1 : 250, Abcam) and rabbit anti‐SOX2 (1/200, Abcam). Subsequently, cells were incubated for 1 h with AlexaFluor‐568‐conjugated secondary antibody (1 : 400, Thermo) in 0.01 m phosphate‐buffered saline (PBS, Gibco, Grand Island, NY, USA) at room temperature. After washing cells with PBS for three times (5 min), the nuclei were counterstained with 4′,6‐diamidino‐2‐phenylindole (DAPI, 1 : 500, Sigma, Saint Louis, MO, USA) in PBS for 10 min at room temperature. Finally, images were captured at 200× magnification using a Zeiss Axio Imager Z2 microscope (Zeiss, Oberkochen, Germany) that was equipped with tissuefaxs software (TissueGnostics GmbH, Vienna, Austria) and prepared using imagej software (Version 1.52a, NIH, Bethesda, MD, USA) and prism 8 for macOS (Version 8.4.0, GraphPad Software, San Diego, CA, USA).

### Flow cytometry of H9‐ESCs

Four days H9‐ESCs were dissociated into single cell with Accutase (STEMCELL Technology) after washing twice with PBS. Then, cells suspension at a concentration of 1 × 10^6^/100 μL (a test) were incubated with Fixable Viability Stain 510 (FVS510, BD Biosciences, 0.1 μL per test) and Alexa Fluor 647 Mouse anti‐SSEA4 monoclonal antibody (BD Biosciences, Franklin Lake, NJ, USA, 2 μL per test) and anti‐TRA‐1‐60‐PE antibody (Miltenyi Biotec, Bergisch Gladbach, Germany, 1 μL per test) for 30 min at 4 °C. After incubation, cells were washed two times with PBS and then filtrated to flat 96‐well plate (300 μL per test). Flow cytometry was performed on Luminex Guava easyCyte (Millipore, Billerica, MA, USA). And flowjo software (Version 10.4, Ashland, OR, USA) was used to analyse data.

### Alkaline phosphatase (AP) staining of H9‐ESCs

To get a better look at colonies of H9‐ESCs, AP staining was used. In brief, cells were fixed with 4% PFA for 30 min at room temperature and then incubated with AP staining solution using BCIP/NBT AP Color Development Kit (C3206, Beyotime, Shanghai, China) for 10 min at dark according to the manufacturer's protocol. Finally, discard the staining solution on the cell surface and add PBS to stop staining. Images were captured using a Nikon fluorescence microscopy (Ti S, Nikon Corporation, Tokyo, Japan) that was equipped with nis‐elements software (Version D4.30.00, Nikon Corporation).

### Three‐dimensional culturing for inducing neural retina (NR) formation

**Fig. 1:**
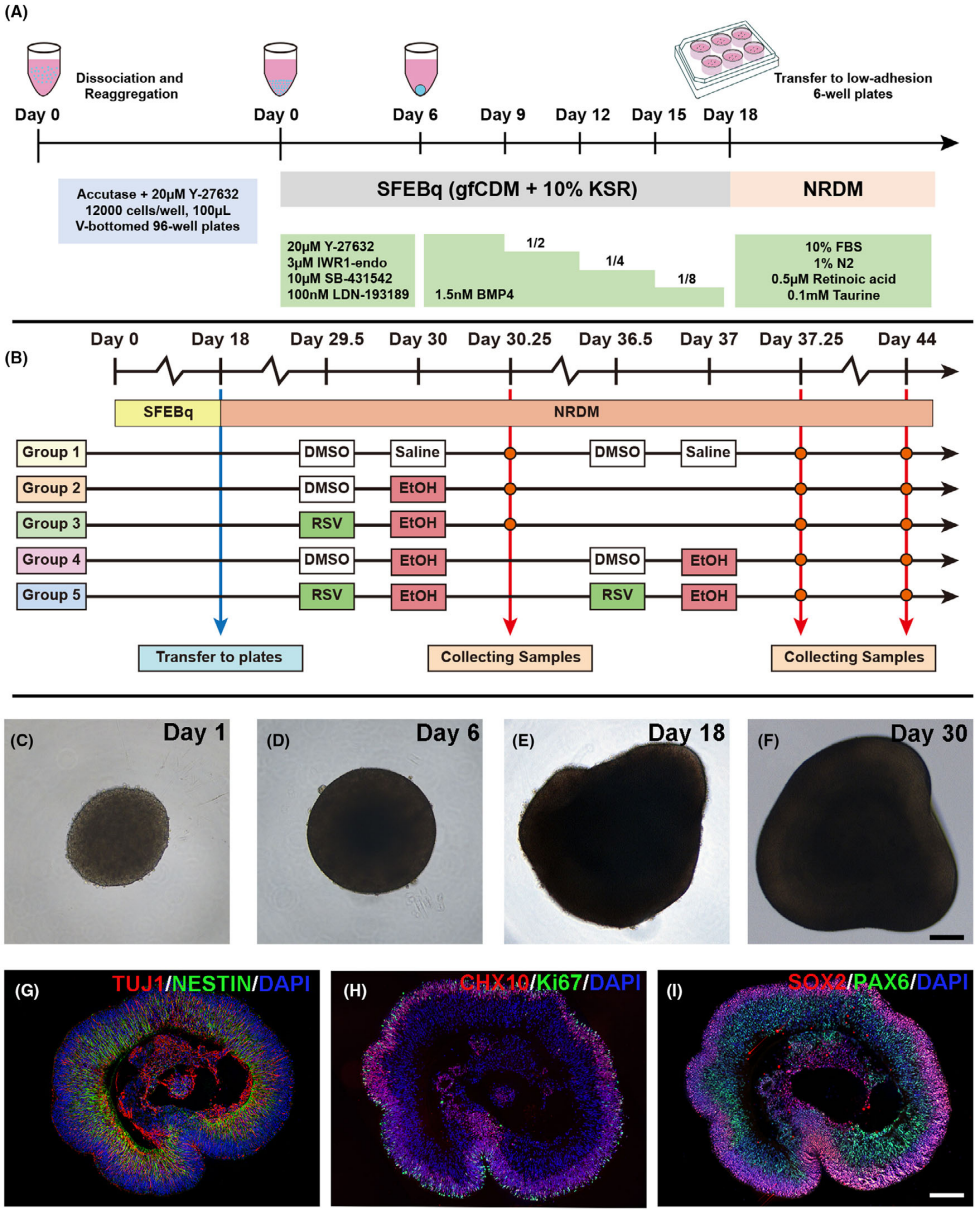
Generation of retinal organoids based on the modified culture method and subsequent experiment design. (A, B) The schematic diagram demonstrates the detailed manipulation of the modified SFEBq culture method for retinal organoids production (A) and the experimental design of our subsequent study (B). BMP4, bone morphogenetic protein 4; DMSO, dimethyl sulfoxide; EtOH, ethanol; FBS, fetal bovine serum; gfCDM, growth factor‐free chemically defined medium; KSR, knockout serum replacement; NRDM, neural retina differentiation medium; RSV, resveratrol; SFEBq, serum‐free floating culture of embryoid body‐like aggregates with quick reaggregation. (C–F) The representative brightfield microscopic images of cultured organoids were taken at 40× magnification on Day 1 (C), Day 6 (D), Day 18 (E) and Day 30 (F), and the peripheral semitransparent part indicates the generated neural retina. Scale bar: 100 μm. (G–I) Representative epifluorescence images (magnified at 200×) of organoid sections on day 44 immunofluorescently stained for TUJ1 and NESTIN (G), CHX10 and KI67 (H), SOX2 and PAX6 (I). Cell nuclei were stained with DAPI (blue). White scale bar: 100 μm.

Three‐dimensional induction was performed according to the SFEBq method with several modifications [33]. In detail, hESCs were dissociated into single cells using Accutase containing 20 μm Y‐27632 for 3 min at 37 °C. After centrifugation (200 ***g*** for 5 min) and resuspension, 1.2 × 10^5^ single hESCs were reaggregated in a V‐bottomed 96‐well conical plate (PrimeSurface; Sumitomo Bakelite, Tokyo, Japan) with 100 μL growth factor‐free chemically defined medium (gfCDM) supplemented with 10% knockout serum replacement (KSR, Gibco), 20 μm Y‐27632, 3 μm IWR1‐endo, 10 μm SB‐431542 and 100 nm LDN‐193189; gfCDM itself contained 45% Iscove's modified Dulbecco's medium‐GlutaMAX (Gibco), 45% Ham's F12‐GlutaMAX (Gibco), 1% chemically defined lipid concentrate (Gibco) and monothioglycerol (450 mM, Sigma). The initiation of hESC plating was defined as day 0. On Day 6, 1.5 nM (55 ng·mL^−1^) recombinant human bone morphogenetic protein 4 (BMP4, R&D, Minneapolis, MN, USA) was added to the culture medium. The concentration of BMP4 was serially diluted by changing the medium on Days 9, 12 and 15. On Day 18, embryonic bodies were transferred into a low‐adhesion 6‐well plate supplemented with NR differentiation medium containing DMEM/F12‐Glutamax medium (Gibco), 1% N2 supplement (Gibco), 10% fetal bovine serum (FBS, Gibco), 0.5 mM retinoic acid (Sigma) and 0.1 mM taurine (Sigma). The culture medium was changed every 3 days (Fig. 1A). Four 96‐well plates were induced in the same passage H9 ESCs, and the success rate of NR induction with the SFEBq method was ~ 90% as defined on Day 30. All differentiation experiments were repeated at least three times on different passages of H9 ESCs to ensure that the experimental data were reproducible and reliable.

### Measurement of retinal organoid (RO) volume

On Days 30, 37 and 44, a total of 15 ROs from three experiments were randomly selected from each group and photographed at 100× magnification using a Nikon Digital Camera (DS‐Ri2, Nikon Corporation). Brightfield images were obtained using an inverted microscopy (Ti S, Nikon Corporation). To achieve better resolution, pictures of different parts of the same RO were stitched together to gain an overall view at high magnification using adobe photoshop cc (Version 20.0.2, Adobe systems incorporated, San Jose, CA, USA) for further analysis. As ROs generally grow into an elliptical or roughly elliptical shape, we regarded RO as an ellipsoid with a long axis (a) and two identical short axes (b). The RO volume is expressed as follows:

$$V_{\mathrm{RO}}=\frac{4}{3}\mathrm{\pi a}b^{2}.$$

### EtOH exposure and RSV pretreatment protocol

EtOH treatment was performed according to a previous study with slight modifications [34]. Briefly, 50 mm EtOH was added to the culture medium for 6 h on Days 30 and 37. An equal volume of saline was added to the culture medium in the corresponding control group. The groups in which EtOH was added singly on day 30 and on both Day 30 and Day 37 were denoted as EtOH1 and EtOH2, respectively. Thus, EtOH1 refers to single alcohol exposure, while EtOH2 refers to two instances of alcohol exposure with a 1‐week interval. Both the EtOH and control groups were sealed with Parafilm™ during the 6‐h EtOH treatment to prevent the evaporation of EtOH. A concentration of 50 mm in culture medium is equivalent to a blood alcohol concentration (BAC) of 230 mg·dL^−1^, which has been shown to significantly influence neurogenesis [34]. After 6‐h EtOH treatment, the culture medium was completely changed to remove EtOH. RSV was added as a pretreatment as described in our previous study [35]. RSV (dissolved in DMSO, Sigma) was added to the culture medium 12 h prior to EtOH treatment to obtain a final concentration of 5 μm. An equal volume of DMSO was added to the culture medium of the corresponding control group. The untreated group was defined as the control group. The group in which RSV was added singly on day 30 was defined as RSV + EtOH1, and that in which it was added on both day 30 and day 37 as RSV + EtOH2. EtOH treatment was applied to EtOH1 and EtOH2 equally to RSV + EtOH1 and RSV + EtOH2. All samples were collected on Day 30 in the control, EtOH1 and RSV + EtOH1 groups, and on Days 37 and 44 in all groups (Fig. 1B).

### Fixation, sectioning and immunofluorescence analysis of NRs

On Day 30, NRs were randomly chosen from the control, EtOH1 and RSV + EtOH1 groups (*N* = 9 from three experiments). On Days 37 and 44, NRs were randomly chosen from the control, EtOH1, RSV + EtOH1, EtOH2 and RSV + EtOH2 groups (*N* = 9 from three experiments). To preserve tissue structure, paraffin embedding of the NRs was performed as previously described [36]. In brief, after washing in 0.01 m PBS three times, NRs were put into 4% PFA in 0.01 m PBS for 12 h for fixation. Fixed NRs were dehydrated with a graded series of ethanol and xylene and then embedded in paraffin wax. Using a paraffin microtome (Leica, Weztlar, Germany), 5‐μm thick paraffin sections were collected. After drying for 2 days at 37 °C, the sections were stored at room temperature. Before performing immunofluorescence analysis, three sections which cross the central part of the NR were selected under a microscope. After deparaffinisation and rehydration, immunofluorescence of the sections was performed as described previously [37]. Briefly, after treatment with 0.2% Triton X‐100 and 10% donkey serum and incubation at room temperature for 30 min, sections were then incubated with the following primary antibodies: anti‐KI67 (1 : 200; Abcam), anti‐PAX6 (1 : 250; Abcam), anti‐SOX2 (1 : 200; Abcam), anti‐β tubulin III (1 : 250; Beyotime), anti‐CHX10 (1 : 200; Santa Cruz, Dallas, TX, USA), anti‐BRN3 (1 : 50; Santa Cruz) and anti‐NESTIN (1 : 200; Sigma) in 10% donkey serum (overnight, 4 °C). The secondary antibodies, Alexa Fluor 488‐ or 568‐conjugated (Thermo), were then applied (1 : 400; 1 h, 25 °C). The terminal deoxynucleotidyl transferase‐mediated dUTP nick end labelling (TUNEL) assay was performed using the *in situ* Cell Death Detection Kit (Fluorescein, Roche, Basel, Schweiz) according to the manufacturer's instructions. In short, sections were treated with 0.2% Triton X‐100 at 37 °C for 60 min and then incubated with working solution (enzyme solution and label solution; 1 : 9 dilution) at 37 °C for 30 min. Before examination using a laser scanning microscope (Zeiss Axio Imager Z2), the sections were counterstained with DAPI (1 : 500, Sigma). The images of TUNEL staining were captured at 1000× magnification with a confocal microscope (Dargonfly 200, ANDOR Technology, Belfast, Northern Ireland) using FITC (green), DAPI (blue) and bright field.

### Cell counting and statistical analysis

In each group, at each time point, at least nine comparable sections from three different organoids crossing the NR centre were selected and stained (*N* = 9 from three experiments). Cell numbers were determined using imagej software (NIH). Specifically, images of the NRs were captured using a high‐magnification microscope (200×). Three 200 μm × 200 μm rectangular areas were randomly chosen from each section. The number of target cells and DAPI‐stained cells in the area was counted manually. More specifically, as SOX2‐ and CHX10‐positive cells are both located in the outer layer of NRs, the ratio of these two markers was calculated to be restricted to the outer layer of NRs. Similarly, PAX6‐positive cells were located in the inner layer of NRs, so the ratio of PAX6‐positive cells was calculated to be restricted to the inner layer of NRs. For TUJ1^+^ cell counting, three 100 μm × 100 μm rectangular areas of each section were randomly chosen at the inner layer of the NRs. TUJ1‐positive cells were counted manually. For statistical analysis, the cellular data were analysed using statistical product and service solutions software V22.0 (SPSS, Chicago, IL, USA). Significance analysis was carried out using one‐way analyses of variance (ANOVA) followed by Fisher's protected least significant difference *post hoc* tests. All data are expressed as the mean ± standard deviation. The significance level was set at *P* < 0.05.

### RNA extraction and subsequent next‐generation RNA sequencing

Five sets of samples collected on day 44 were used for RNA extraction, with three biological replicates per set. Five organoids from three different batches were selected from each group (*N* = 15 from three experiments). The total RNAs of the organoids were extracted using TRIzol reagent (Invitrogen, Carlsbsd, CA, USA) according to the manufacturer's instructions. RNA concentrations were initially assessed using a NanoDrop 2000 (*Nanodrop*, Thermo Fisher, Waitham, MA, USA) and were further precisely determined along with the qualification assessment using a Bioanalyzer 2100 (Agilent Technologies, Colorado Springs, CO, USA) and TapeStation 4200 system (Agilent). cDNA library construction was performed based on qualified RNA samples using the VAHTS mRNA‐seq v2 Library Prep Kit (Vazyme NR611‐02, Nanjing, China). After qualification using the Qubit 3.0 (Thermo), cDNA libraries of each group were loaded onto the Illumina NovaSeq 6000 (6X Genomics, Illumina, lnc, San Diego, CA, USA) for sequencing with paired‐end reads (PE150). Each group was sequenced in triplicate. Next, reads with *N* > 3 or adapters and low‐quality reads in the sequenced raw data were filtered out, and the remaining reads were aligned to the Silva database using bowtie2 software (Version 2.4.5) to filter out the rRNAs [38]. Finally, the clean reads were aligned to the human genome (GRCh38.p12) using hisat2 (version 2.2.1) to generate a gene expression matrix [39].

### Data analysis of RNA sequencing

The comparison between RSV + EtOH1 and EtOH1 was termed comparison A (RSV + EtOH1 vs ETOH1) and that between RSV + EtOH2 and EtOH2 was termed comparison B (RSV + EtOH2 vs EtOH2). Differentially expressed genes (DEGs) were determined using the ‘edger’ r package (version 3.39.1) [40, 41, 42] with the criterion of *P* < 0.05. Next, the ‘clusterprofiler’ (version 4.5.0) [43, 44] and ‘org.Hs.eg.db’ [45] r package were used for enrichment analyses of gene ontology (GO) and Kyoto Encyclopedia of Genes and Genomes (KEGG) pathways, as well as gene set enrichment analysis (GSEA) for the differentially expressed genes. The analysis results were visualised using the r packages of ‘enrichplot’ [46] (https://yulab‐smu.top/biomedical‐knowledge‐mining‐book/) and ‘pheatmap’ (version 1.0.12) [47].

### Real‐time quantitative polymerase chain reaction (RT‐qPCR)

After total RNAs were extracted, reverse transcription‐polymerase chain reaction (RT‐PCR) was performed to synthesise cDNA using a Toyobo reverse transcription kit (Toyobo, Osaka, Japan) and then followed by RT‐qPCR for the genes of interest using Toyobo PCR Master Mix (Toyobo) on the CFX Manager system (version 3.1, Bio‐Rad Laboratories, Hercules, CA, USA) according to the manufacturer's instructions. The primers used for the target genes are listed in Table 1. A two‐tailed Student's *t*‐test was used to compare the PCR results. *P* < 0.05 was considered statistically significant.

**Table 1:** Primer sequences used for quantitative real‐time PCR.

Gene symbol	5′‐forward primer sequence‐3′	5′‐reverse primer sequence‐3′
RELN	CACAATGCTCTCTCCTCCCG	TGCCAGGAATCCGATCTTGC
SGK1	TGGCACGCCGGAGTATCT	TGGGTTAAAAGGGGGAGTAATCTT
FGFR2	GACCAAACGTATCCCCCTGC	TTGCCCAGTGTCAGCTTATCT
MET	TGGGCACCGAAAGATAAACCT	TCGGACTTTGCTAGTGCCTC
PIK3AP1	ATGGCAGCCTCAGGGGT	ACTGGACAGGAACAGGGTCT
GAPDH	ATTGCCCTCAACGACCACT	ATGAGGTCCACCACCCTGT

## Results

### NR generation from H9 hESC cell line and subsequent EtOH treatment

Using feeder‐free culture methods, H9‐ESCs proliferated well and displayed a classical round shape and a clone‐like growth (Fig. S1A–C). Immunofluorescence showed that H9‐ESCs expressed stemness markers such as NANOG, SOX2 and OCT4 (Fig. S1D–L). All positivity rates for these three markers were over 95% (Fig. S1M). H9‐ESCs also expressed pluripotent stem cell surface markers SSEA4 and TRA‐1‐60 (Fig. S1N). Moreover, clones of H9‐ESCs were variably stained with alkaline phosphatase and displayed a light and homogenous staining pattern (Fig. S1O). These results showed that H9‐ESCs exhibit all the characteristics of embryonic stem cells. Using modified SFEBq culturing methods (Fig. 1A), hESCs gathered to form round‐shaped embryoid bodies (EBs) (Fig. 1C). After culturing in SFEBq medium for 6 days, all EBs grew into a circle on Day 6, when BMP4 was added to the medium (Fig. 1D). After addition and subsequent sequential half‐dilution of BMP4, the EBs gradually sprouted NRs (Fig. 1E). At the time of RSV pretreatment and EtOH treatment (Day 30), over 95% of the EBs formed irregular oval shapes with a continuous semitransparent epithelium (Fig. 1F). After culturing in NR differentiation medium (NRDM) for 2 weeks, the EBs gradually differentiated into NRs that contained TUJ1‐, NESTIN‐, CHX10‐, KI67‐, SOX2‐ and PAX6‐positive cells (Fig. 1G–I). On Days 30 and 37, EtOH treatment was implemented after 12 h of RSV pretreatment. The samples were collected on Days 30, 37 and 44 (Fig. 1B). The volume of the ROs was calculated at the point of sample collection. The results showed that the volume of the ROs increased with time (Fig. S2). Although not significant, the volumes of EtOH‐treated ROs were mostly smaller than those of control ROs (Fig. S2A,B,D,E,G,I,J,L). This effect was more evident in the double EtOH treatment group on Day 44. RSV pretreatment partially prevented the situation caused by the first and second EtOH exposures (Fig. S2C,E–H,J–M). However, all the volume comparisons were insignificant (Fig. S2N–P, *P* > 0.05).

### RSV improved the proliferation of NRs following alcohol treatment

**Fig. 2:**
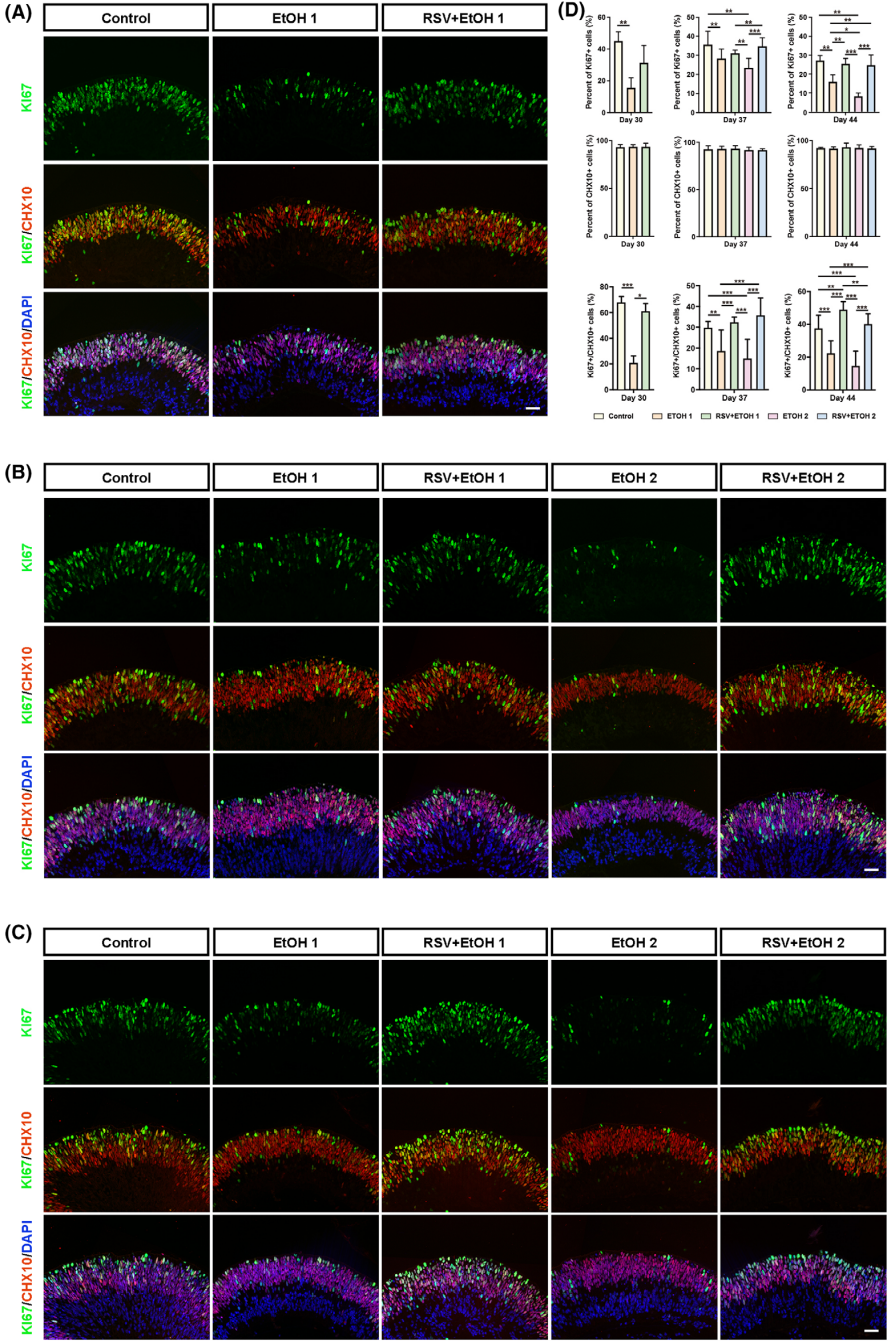
Expression differences concerning KI67 and CHX10 under immunofluorescence in each organoid group at three time points. (A–C) Representative immunofluorescent images of organoid sections stained for KI67 (green) and CHX10 (red) were taken at 200× magnification at day 30 (A), Day 37 (B) and Day 44 (C). Nuclei were stained with DAPI (blue). Scale bar: 40 μm. (D) Comparison of the percentages of cells positive for KI67 and CHX10 among groups at each time point by one‐way ANOVA analysis and Bonferroni's *post hoc* test (*N* = 9 from three experiments). Error bars represent standard deviations. EtOH, ethanol; RSVm resveratrol. *P < 0.05, **P < 0.01, ***P < 0.001.

To identify the effect of alcohol on early retinal development, we performed CHX10 and KI67 immunofluorescence analysis. The results showed that KI67‐ and CHX10‐positive cells were mainly located in the outer layer and outside the border of NRs, respectively. There was no significant difference in the number of CHX10‐positive cells among all groups at any of the three time points (*P* > 0.05) (Fig. 2). However, the number of KI67‐positive cells within the NRs decreased significantly after treatment with 50 mm EtOH on day 30 (EtOH1) compared with that in the control group (control) (*P* < 0.01) (Fig. 2A,D). This significant influence was sustained for 1 and 2 weeks (Day 37 and Day 44) after a single EtOH exposure (*P* < 0.01, *P* < 0.01) (Fig. 2B–D). The second 50 mM EtOH exposure (EtOH2) also led to a significant decrease in the number of KI67‐positive cells on days 37 and 44 compared with the KI67‐positive cells in the control (*P* < 0.01, *P* < 0.01) (Fig. 2D). In addition, the number of KI67‐positive cells was lower in EtOH2 than in EtOH1. The decrease was insignificant on day 37 but significant on day 44 (Fig. 2D) (*P* > 0.05, *P* < 0.05). Pretreatment with RSV before the first exposure to EtOH (RSV + EtOH1) improved the rate of KI67‐positive cells on Days 30 and 37, but with significance on day 44 (Fig. 2D). However, double pretreatments with RSV before exposure to EtOH (RSV + EtOH2) significantly improved the rate of KI67‐positive cells on both day 37 and day 44 (*P* < 0.001, *P* < 0.001). Similar results were found in KI67‐ and CHX10‐positive cells. Single or double alcohol exposure resulted in a decrease in the number of KI67‐ and CHX10‐positive cells on Days 30, 37 and 44 (all *P* < 0.05), and pretreatment with RSV reduced the damage caused by 50 mm EtOH.

### RSV suppressed the apoptosis of NRs following alcohol treatment

**Fig. 3:**
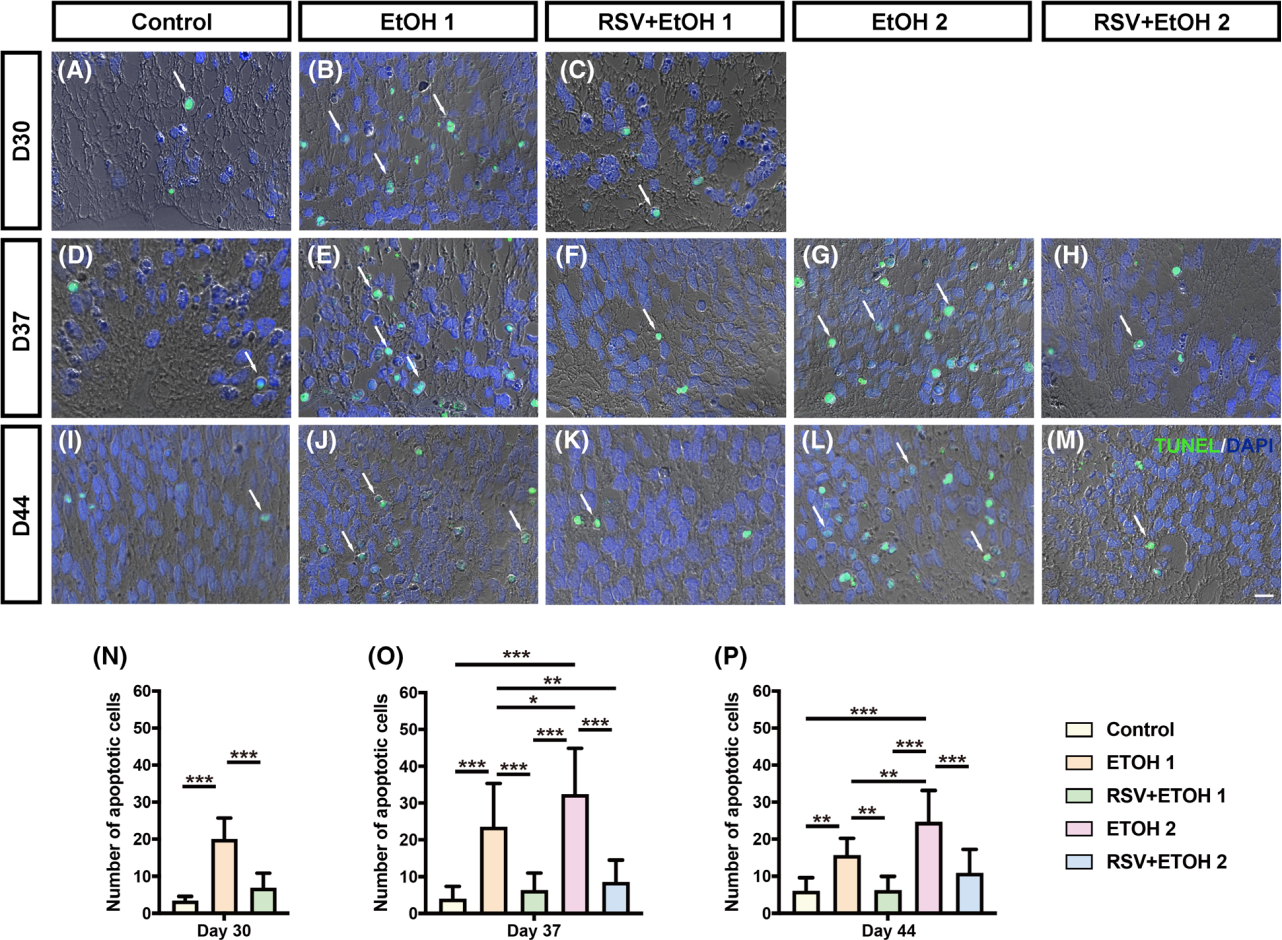
Cell apoptosis in the neural retinal induced by alcohol treatment was mitigated by resveratrol. (A–M) Representative fluorescent images of apoptotic cells indicated with TUNEL (green dot for dUTP) were taken at 1000× magnification. Nuclei were stained with DAPI (blue). White arrows indicate co‐stained cells (apoptotic cells). Scale bar: 10 μm. (N–P) Comparative analysis of numbers of apoptotic cells among groups at each time point by one‐way ANOVA analysis and Bonferroni's *post hoc* test (*N* = 9 from three experiments). Error bars represent standard deviations. EtOH, ethanol; RSV, resveratrol. *P < 0.05, **P < 0.01, ***P < 0.001.

TUNEL immunofluorescence analysis was performed to test the apoptosis‐inducing effect of alcohol on early retinal development. The results showed that TUNEL^+^ apoptotic cells were sparsely distributed in the inner nuclear layer of NRs (Fig. S3). Exposure to EtOH alone significantly increased the number of apoptotic cells in NRs on Day 30 (Fig. 3A–B,N) (*P* < 0.001), an effect that persisted on Days 37 and 44 (Fig. 3D–E,I–J,O–P) (*P* < 0.001, *P* < 0.01). The second exposure to EtOH further increased the number of apoptotic cells on Day 37 (Fig. 3E,G,O) (*P* < 0.05). This effect was also observed on Day 44 (Fig. 3J,L,P) (*P* < 0.01). The pretreatment with RSV before the first exposure to EtOH instantly decreased the number of apoptotic cells in NRs on Day 30 (Fig. 3B–C,N) (*P* < 0.001). This apoptosis‐suppressing effect was also significant on Days 37 and 44 (Fig. 3E–F,J–K,O–P) (*P* < 0.001, *P* < 0.01). Moreover, pretreatment with RSV before exposure to EtOH (RSV + EtOH2) also presented a significant apoptosis‐suppressing effect on Day 37, compared with that seen in the double EtOH exposure group (EtOH2) (Fig. 3G–H,O) (*P* < 0.001). The number of apoptotic cells in the RSV + EtOH2 group was still significantly lower than that in EtOH2 on day 44 (Fig. 3L–M,P) (*P* < 0.001).

### RSV protected PAX6 ^+^ retinal progenitor cells from alcohol‐induced damage

**Fig. 4:**
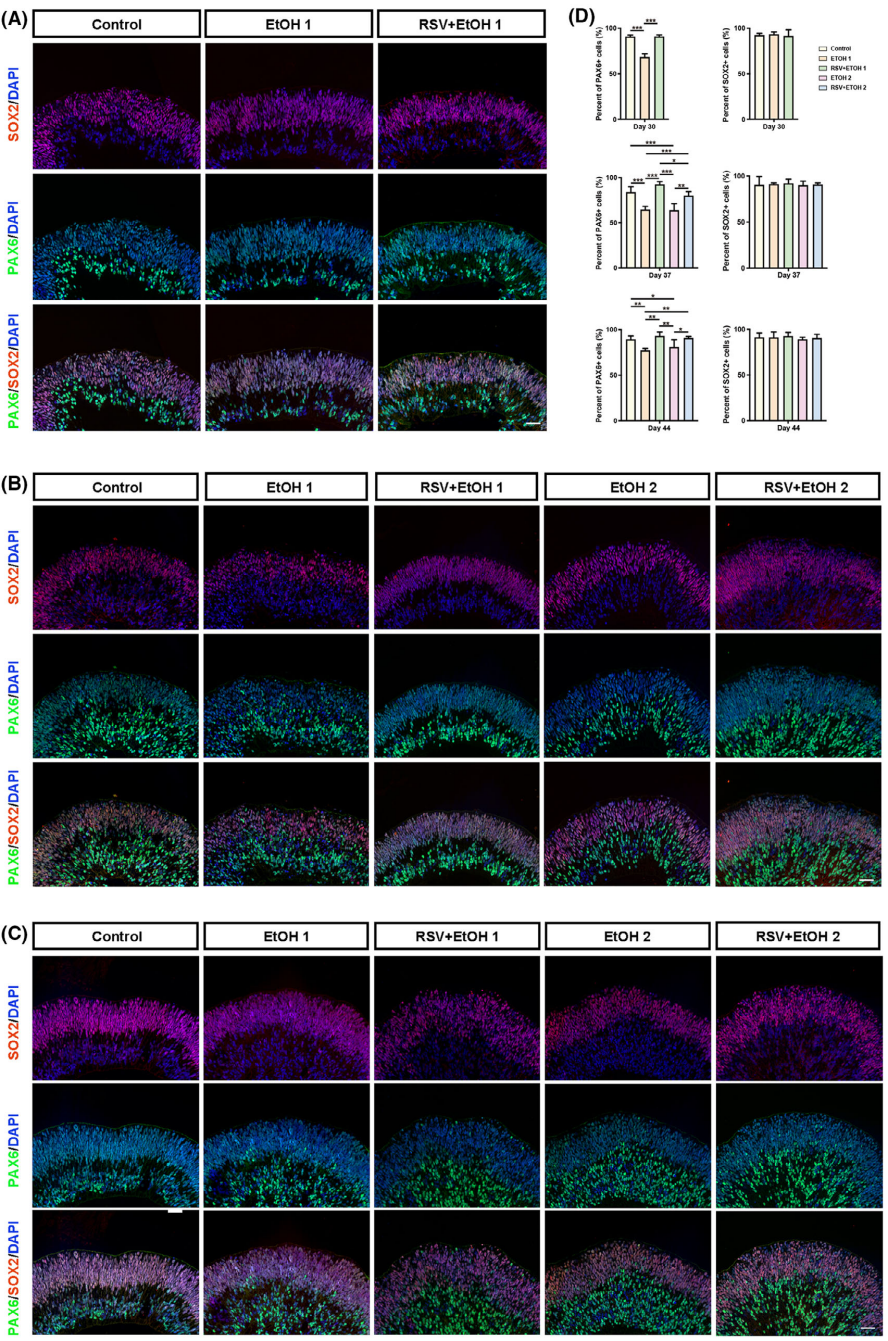
Resveratrol protected PAX6‐positive retinal progenitor cells from alcohol damage. (A–C) Representative immunofluorescent images of organoid sections stained for PAX6 (green) and SOX2 (red) in each group at Day 30 (A), Day 37 (B) and Day 44 (C) were taken at 200× magnification. Notably, PAX6‐ and SOX2‐positive cells were mainly located in the inner layer and outer layer of organoids, respectively. Nuclei were stained with DAPI (blue). Scale bar: 40 μm. (D) Comparison of percentages of cells positive for PAX6 and SOX2 among groups at three time points by one‐way ANOVA analysis and Bonferroni's *post hoc* test (*N* = 9 from three experiments). Error bars represent standard deviations. EtOH, ethanol; RSV, resveratrol. *P < 0.05, **P < 0.01, ***P < 0.001.

To further determine how alcohol influences retinal development, we performed PAX6 and SOX2 double‐immunofluorescence analyses. The results showed that PAX6‐ and SOX2‐positive cells were mainly located in the inner and outer layers of the NRs, respectively (Fig. 4). There was no significant difference in the number of SOX2‐positive cells among all groups at any of the three time points (*P* > 0.05). However, the rates of PAX6‐positive cells instantly decreased significantly after single and double EtOH exposure on Day 30 and Day 37, respectively (Fig. 4A,B) (*P* < 0.001, *P* < 0.001). The PAX6^+^ cell‐decreasing effect of a single EtOH exposure persisted on Days 37 and 44 (*P* < 0.001, *P* < 0.01), and this effect of double EtOH exposure persisted to Day 44 (Fig. 4B,C) (*P* < 0.05). However, no significant difference was found between the two EtOH exposure groups on either Day 37 or Day 44 (Fig. 4D) (*P* > 0.05, *P* > 0.05). The pretreatment with RSV before the first exposure to EtOH significantly improved the number of PAX6‐positive cells on Day 30 (*P* < 0.001). This effect lasted until Days 37 and 44 (*P* < 0.001 and *P* < 0.01, respectively). Double pretreatments of RSV before two exposures to EtOH significantly increased the rates of PAX6‐positive cells on both Day 37 and Day 44 (*P* < 0.01, *P* < 0.05). In addition, differences among different groups became narrower over time (Fig. 4D).

### RSV promoted the neurogenesis of RGCs damaged by alcohol

**Fig. 5:**
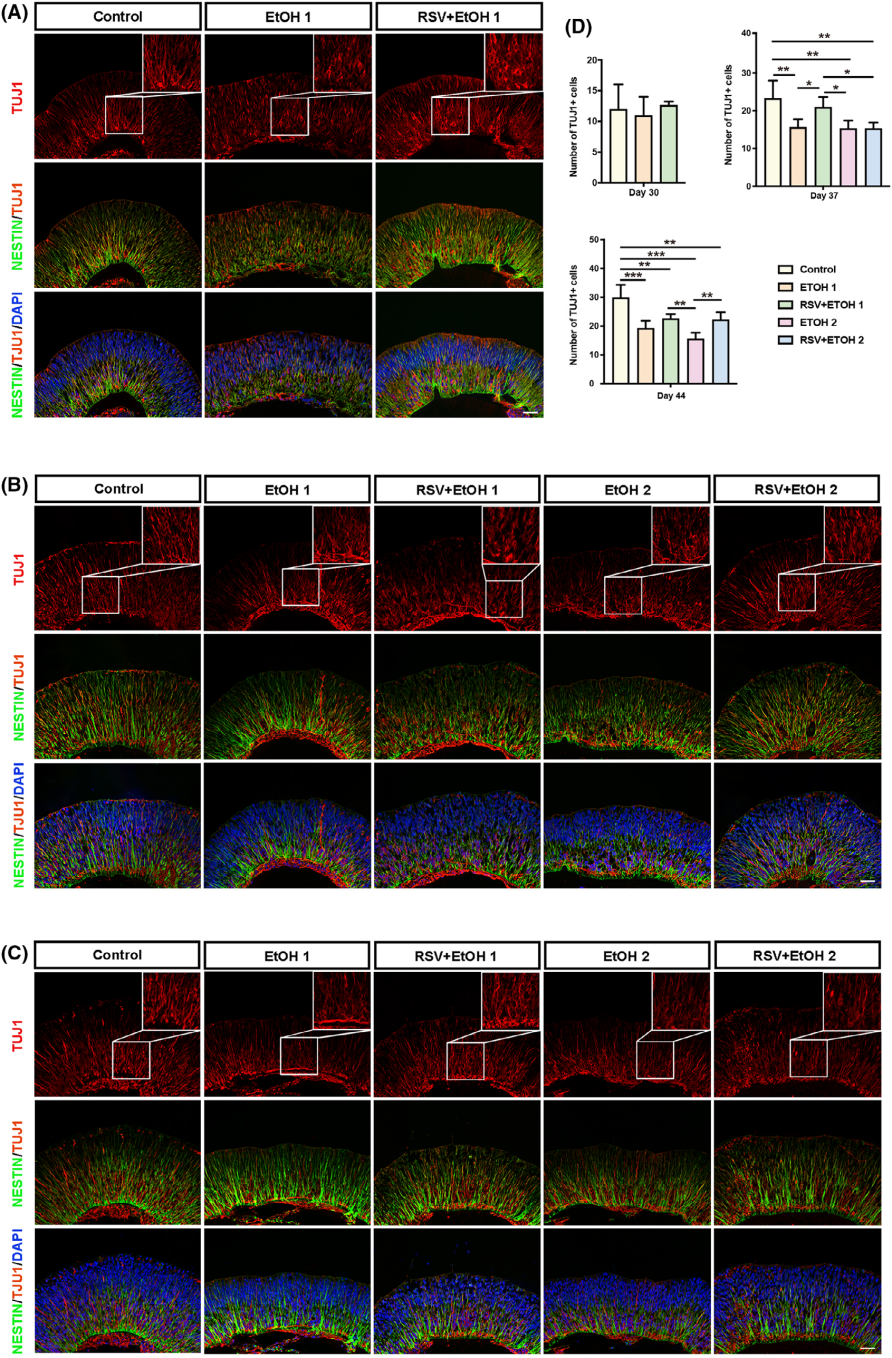
Resveratrol prevented alcohol‐induced damage of TUJ1^+^ retinal ganglion cells neurogenesis. (A–C) Representative immunofluorescent images of organoid sections stained for NESTIN (green) and TUJ1 (red) in each group at each time point were taken at 200× magnification at Day 30 (A), Day 37 (B) and Day 44 (C). Inset shows magnified view (400×) of the rectangular region in the TUJ1 layer. Nuclei were counterstained with DAPI (blue). TUJ1 was used to mark ganglion cells and NESTIN for the retinal stem cells. Scale bar: 40 μm. (D) Comparison of rates of TUJ1^+^ cells among groups at each time point by one‐way ANOVA analysis and Bonferroni's *post hoc* test (*N* = 9 from three experiments). Error bars represent standard deviations. EtOH, ethanol; RSV, resveratrol. *P < 0.05, **P < 0.01, ***P < 0.001.

**Fig. 6:**
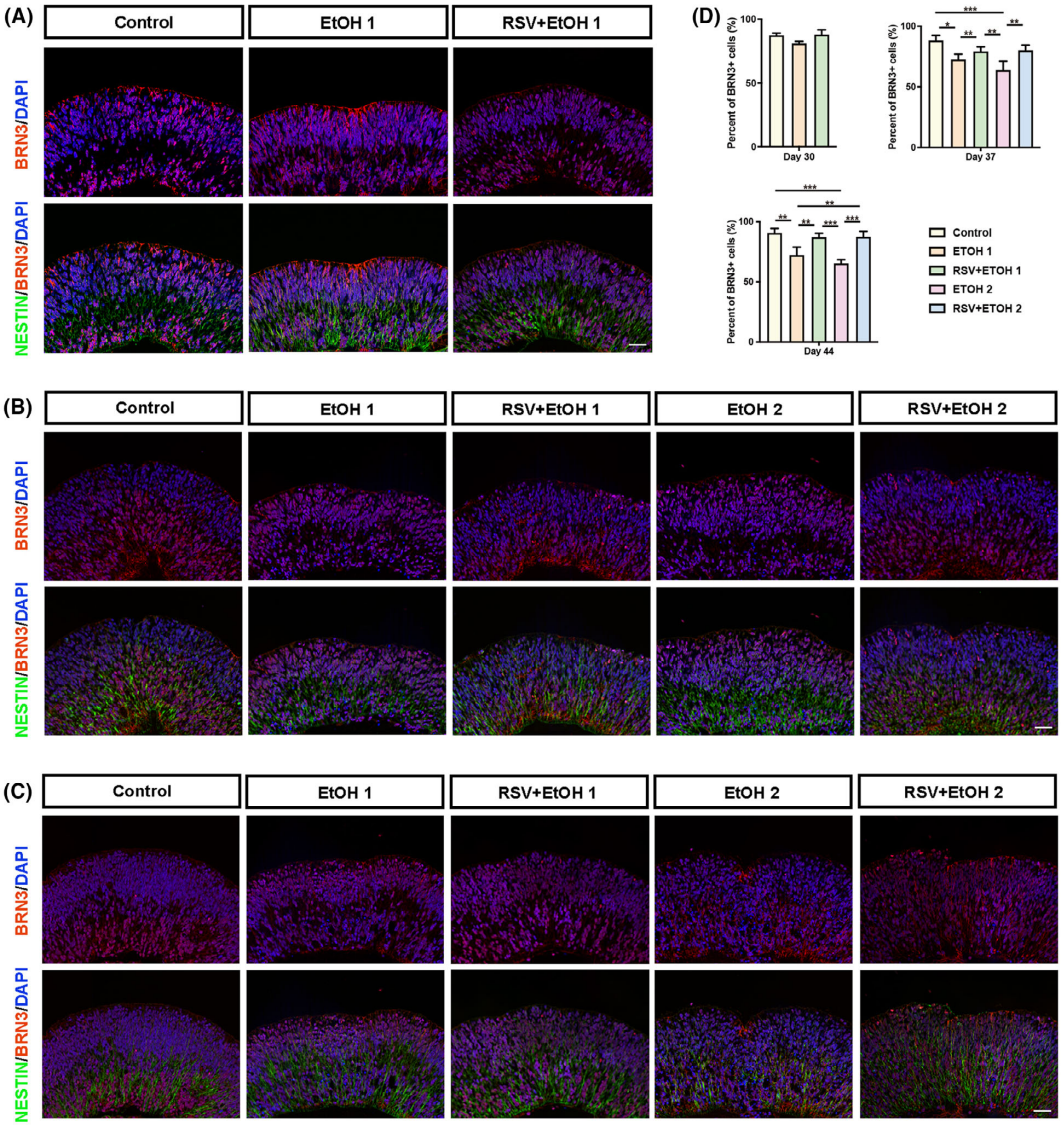
Resveratrol protected BRN3‐positive retinal ganglion cells from alcohol damage. (A–C) Representative immunofluorescent images of organoid sections stained for NESTIN (green) and BRN3 (red) in each group at each time point were taken at 200× magnification at Day 30 (A), Day 37 (B) and Day 44 (C). Nuclei were counterstained with DAPI (blue). Scale bar: 40 μm. (D) Comparison of rates of BRN3^+^ cells among groups at each time point by one‐way ANOVA analysis and Bonferroni's *post hoc* test (*N* = 9 from three experiments). Error bars represent standard deviations. EtOH, ethanol; RSV, resveratrol. *P < 0.05, **P < 0.01, ***P < 0.001.

The influence of alcohol on early RGCs was also analysed. TUJ1 and BRN3 were used to mark differentiated RGCs, as described in our previous study [37]. To better represent the migration of RGCs, the neural stem cell marker NESTIN was used to mark the retinal stem cells, which act as scaffolds to guide the migration of RGCs. Our previous study confirmed that cell proliferation occurs at the apical side, and newly generated RGCs migrate inwards to the basal side of the retinal organoids [37]. By performing TUJ1 and NESTIN double staining, we found that a single EtOH exposure did not immediately induce a significant difference in the number of agminated TUJ1^+^ cells at the basal side on day 30 (Fig. 5A,D) (*P* > 0.05). However, 1 week later, on Day 37, the influence of the single EtOH exposure started to emerge, as the number of TUJ1^+^ cells was significantly reduced in EtOH1 compared with that in the control (Fig. 5B,D) (*P* < 0.01). This persisted on Day 44 (Fig. 5C,D) (*P* < 0.001). The double EtOH exposure did not show an immediate influence compared with the single EtOH exposure on Day 37 (*P* > 0.05). However, double EtOH exposure further reduced the number of TUJ1^+^ cells on Day 44, although the difference was not statistically significant (*P* > 0.05). RSV pretreatment did not present an instant effect on EtOH exposure (*P* > 0.05). However, 1 week after EtOH treatment, the number of TUJ1^+^ cells both after single and double EtOH exposure on Day 37 and Day 44, respectively, were significantly promoted in the pretreated groups (*P* < 0.05, *P* < 0.01). Double staining with BRN3 and NESTIN showed the same results. On Day 30, the number of BRN3‐positive ganglion cells did not differ significantly among the three groups (Fig. 6A,D) (*P* < 0.05). Alcohol exposure significantly inhibited the number of BRN3‐positive cells compared with the BRN3‐positive cells in the control on Days 37 and 44 (Fig. 6B–D) (all *P* < 0.05). RSV pretreatment prevented alcohol‐induced reduction of BRN3‐positive cells on Days 37 and 44 (Fig. 6B–D) (all *P* < 0.01).

### Different gene expression profiles and associated GO terms of EtOH‐afflicted NRs with or without RSV administration

**Fig. 7:**
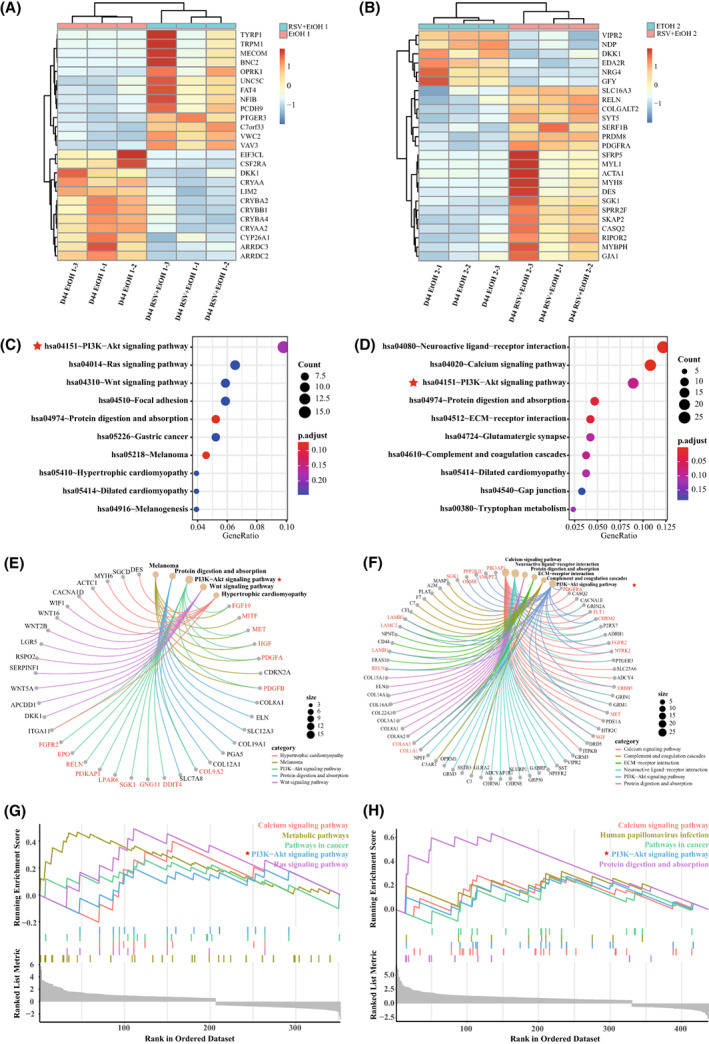
Enrichment analysis of the differentially expressed genes involved in resveratrol action in alcohol‐burdened retinal organoids at Day 44. (A, B) The top 25 differentially expressed genes in RSV + EtOH1 vs. EtOH1 (A) and RSV + EtOH2 vs. EtOH2 (B). Dark blue and dark red in the colour scale correspond to the most downregulated genes and the most upregulated genes, respectively. (C, D) The bubble diagrams illustrated the top 10 enriched Kyoto Encyclopaedia of Genes and Genomes pathways for the differentially expressed genes in RSV + EtOH1 vs. EtOH1 (C) and RSV + EtOH2 vs. EtOH2 (D). The bubble size corresponds to the number of enriched genes and the colour scale corresponds to the adjusted *P*‐value. (E, F) The network diagrams demonstrate the relationship between five representative pathways and the related top differentially expressed genes in RSV + EtOH1 vs. EtOH1 (E) and RSV + EtOH2 vs. EtOH2 (F). Dot size signifies the number of involved genes. Red dot indicates the gene enriched to the PI3K‐AKT pathway. (G, H) The enrichment plot of GSEA results for the differentially expressed genes in RSV + EtOH1 vs EtOH1 (G) and RSV + EtOH2 vs. EtOH2 (H). The vertical bar denotes individual differentially expressed genes hit in the pathway. The red star highlights the PI3K‐AKT signalling pathway. EtOH, ethanol; RSV, resveratrol.

The cultured retinal organoids were subjected to EtOH once or twice, as described above, to simulate the one‐shot or intermittent alcohol consumption. Parallel cultures with 12 h of RSV pretreatment prior to each alcohol stimulation were performed to explore the effects of RSV on the development of alcohol‐burdened retinas. Using next‐generation sequencing, 363 and 451 DEGs were detected in comparison A (RSV + EtOH1 vs. EtOH1) and comparison B (RSV + EtOH2 vs. EtOH2), respectively. The grouped samples could be easily discriminated by the top 25 DEGs (Fig. 7A,B), which suggested reliable RNA sequencing results. Furthermore, the GO enrichment analyses of the DEGs in the two comparisons were carried out separately; the results showed that the enriched GO terms were more concerned with eye or visual system development in the single treatment group, whereas the GO terms concerning muscle tissue development were most enriched in the double treatment group. The top 10 enriched GO terms with the number of enriched genes in the two comparisons are illustrated separately in the bubble diagrams (Fig. S4A,B). The relationships between five representative GO terms and the enriched top DEGs in the two comparisons are demonstrated with the chord diagrams separately (Fig. S4C,D). The profiles and enrichment analyses of DEGs between RSV + EtOH1 (RSV + EtOH2) and control are shown in Figs S5 and S6.

### RSV activated the PI3K‐AKT signalling pathway in NRs under alcohol stimulation

To ascertain the underlying molecular pathways associated with RSV in the alcohol‐stimulated retinal organoids, we further conducted enrichment analyses of KEGG pathways for the aforementioned DEGs in the two comparisons separately. The results indicated that the top 10 enriched pathways for comparison A included cell proliferation‐related pathways, such as the PI3K‐AKT, RAS and WNT signalling pathways (Fig. 7C), while the top 10 enriched pathways for comparison B were more diverse, such as the neuroactive ligand–receptor interaction, calcium signalling pathway, and PI3K‐AKT signalling pathway (Fig. 7D). Notably, the PI3K‐AKT signalling pathway was involved in both comparisons. The links between the five representative enriched pathways and their associated DEGs in the two comparisons were plotted separately plotted as network diagrams (Fig. 7E,F). Furthermore, GSEA was performed to determine the functional status of the pathways, and the results showed that the calcium signalling pathway, metabolic pathway, pathways in cancer, PI3K‐AKT signalling pathway and RAS signalling pathway were activated in the comparison A (Fig. 7G). Meanwhile, several pathways including the calcium signalling pathway, human papillomavirus infection, pathways in cancer, PI3K‐AKT signalling pathway, protein digestion, and absorption, were activated in comparison B (Fig. 7H). Among the activated pathways, the PI3K‐AKT signalling pathway, a cell survival‐related pathway, was simultaneously activated in the two comparisons, which may suggest its protective role in alcohol‐challenged retinal organoids pretreated with RSV.

### Validation of DEGs associated with the PI3K‐AKT signalling pathway by RT‐qPCR

**Fig. 8:**
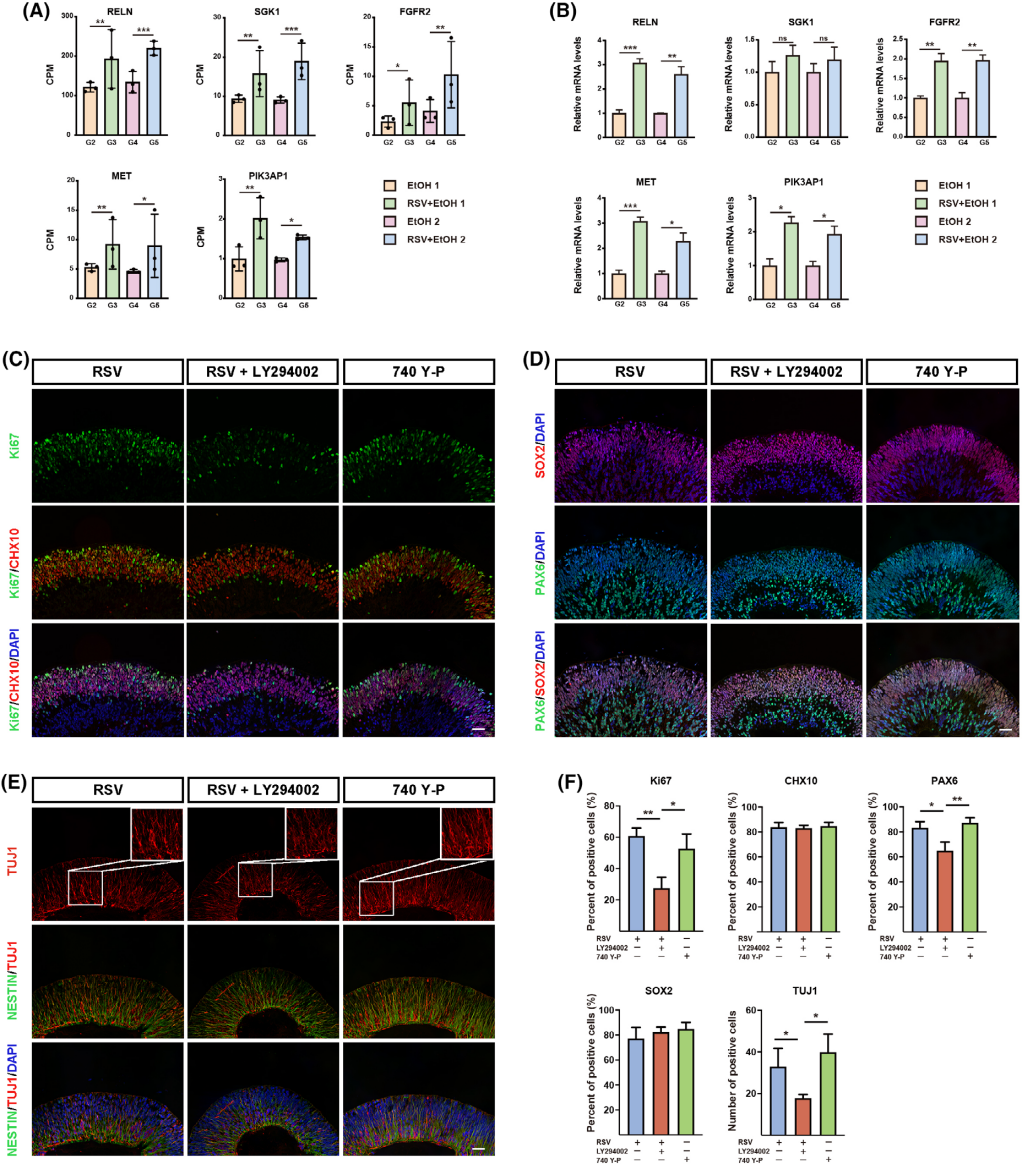
Resveratrol protected the neural retina from alcohol damage by activating the PI3K‐AKT signalling pathway. (A) Counts per million of RNA sequencing were used to plot the expression levels of RELN, SGK1, FGFR2, MET and PIK3AP1. Each dot represents one sample. (B) The relative mRNA levels of RELN, SGK1, FGFR2, MET and PIK3AP1 between the EtOH group and RSV groups by unpaired Student's *t*‐test, normalised by GAPDH. Error bars represent standard error of the mean. (C) Representative immunofluorescent images of organoid sections stained for KI67 (green) and CHX10 (red) in each group at 200× magnification. (D) Representative immunofluorescent images of organoid sections stained for PAX6 (green) and SOX2 (red) in each group at 200× magnification. (E) Representative immunofluorescent images of organoid sections stained for NESTIN (green) and TUJ1 (red) in each group at 200× magnification. (F) Comparison of positive cells among groups by one‐way ANOVA analysis and Bonferroni's *post hoc* test (*N* = 9 from three experiments). Nuclei were counterstained with DAPI (blue). Scar bars: 40 μm. Error bars represent standard deviations. EtOH, ethanol; RSV, resveratrol. *P < 0.05, **P < 0.01, ***P < 0.001, ns, no significance.

To further verify the role of the PI3K‐AKT signalling pathway in the RSV‐mediated reversal of alcohol‐induced NR damage, five DEGs associated with the PI3K‐AKT signalling pathway were selected, including RELN, SGK1, FGFR2, MET and PIK3AP1. These genes were upregulated in both comparisons A and B. Counts per million (CPM) of the five mRNAs detected by the edgeR package in the RSV group were significantly higher than those in the EtOH group of comparison A (*P* = 0.0029, 0.0015, 0.0152, 0.0076 and 0.0028, respectively), and comparison B (*P* = 5.91E‐07, 6.79E‐07, 0.0026, 0.0138 and 0.0479, respectively) (Fig. 8A). To verify the reliability of the RNA sequencing data, the relative expression levels of the five mRNAs in each group were quantified by RT‐qPCR and normalised to GAPDH expression. The results showed that the expression levels of RELN, FGFR2, MET and PIK3AP1 under RSV pretreatment were higher than those under alcohol stimulation alone in comparisons A (*P* = 0.0008, 0.0094, 0.0008 and 0.0112, respectively) and B (*P* = 0.0081, 0.0084, 0.0271 and 0.0283, respectively) (Fig. 8B). Although no statistically significant difference was found in SGK1 expression (*P* = 0.333 in comparison A, 0.4758 in comparison B), there was a trend of increased expression after RSV pretreatment compared with the SGK1 expression level in the alcohol group. These results implied that RSV may activate the PI3K‐AKT signalling pathway to protect the NR from alcohol damage.

### RSV protected NRs from alcohol damage via activating the PI3K‐AKT signalling pathway

To further clarify PI3K‐AKT as the main pathway of RSV protection against alcohol‐induced NR damage, a PI3K‐AKT inhibitor (LY294002) and agonist (740 Y‐P) were used. Three groups were included in this study. Specifically, the control group (RSV treatment) received only 5 μm RSV pretreatment for 12 h. The RSV + LY294002 group was treated with 5 μm RSV and 20 μm LY294002 for 12 h. ROs in the 740 Y‐P group were treated with 20 μm 740 Y‐P for 12 h. All groups were then treated with 50 mM alcohol for another 6 h. The results showed that inhibition of the PI3K‐AKT signalling pathway resulted in a decrease in KI67‐positive cells compared with the KI67‐positive cells seen in the RSV and 740 Y‐P groups (Fig. 8C,F) (*P* < 0.01, *P* < 0.05). Moreover, PAX6‐positive progenitor cells and TUJ1‐positive ganglion cells were also inhibited by LY294002, as there were significantly fewer positive cells after the addition of LY294002 compared with those seen following single RSV treatment (Fig. 8D–F) (*P* < 0.05, *P* < 0.05). Similarly, the percentage of PAX6‐positive cells and the number of TUJ1‐positive cells in the 740 Y‐P group were significantly higher than the corresponding parameters in the RSV + LY294002 group (*P* < 0.01, *P* < 0.05). However, no significant difference was observed between the RSV and 740 Y‐P groups (*P* > 0.05). These results indicated that RSV protects NRs from alcohol damage by activating the PI3K‐AKT signalling pathway.

## Discussion

In the present study, we used retinal organoids to investigate the specific effects of EtOH on retinal development and elucidated the protective role of RSV pretreatment on retinal cells. These results confirmed that EtOH exposure inhibited proliferation and promoted apoptosis of retinal cells. Moreover, EtOH targeted the inner layer of the early developing retina, threatened the survival of retinal progenitor cells and impeded the differentiation of RGCs. However, after pretreatment with RSV, the proliferation‐inhibiting and apoptosis‐promoting effects were partially inhibited. In addition, the survival of retinal progenitor cells and the differentiation of RGCs were improved following RSV pretreatment. To determine the underlying protective mechanism, RNA sequencing and RT‐qPCR were performed. The results showed that activation of the PI3K‐AKT signalling pathway may be the mechanism by which RSV exerted its protective effects. The findings obtained herein provide pathological evidence of retinal involvement in FAS and suggest a potential treatment approach.

To better reflect this fact, we treated retinal organoids once and twice with EtOH, which mimicked single and double alcohol exposure during early pregnancy. We also set the time interval of the two EtOH treatments to 1 week to simulate two separate occurrences of alcohol intakes during two adjacent weekends. Using brain organoid models, a previous study confirmed that EtOH‐induced cell apoptosis within organoids is dose‐dependent. Cells exhibited obvious apoptosis when the EtOH concentration reached 50 mm [34]. In the present study, we used 50 mM EtOH to carry out the experiments, and all culture plates were sealed with parafilm during EtOH treatment. This protocol has already been adopted in several other studies [34, 48]. Using the KI67 antibody, we confirmed that proliferation of human retinal cells was inhibited after EtOH exposure. Repeated exposures further decreased the number of proliferating cells. The proliferation‐inhibiting effect of EtOH during development has already been shown [49, 50, 51]. More specifically, retinal cell proliferation is disrupted after EtOH treatment in zebrafish embryos [52]. In retinal organoids, cells proliferate on the apical side of the neural epithelium [37]. The polarity characteristics of the human retina remained constant following EtOH treatment in our study, which corresponds with previous results in zebrafish [52]. In addition, using the TUNEL assay, we found that apoptosis of human retinal cells increased following EtOH exposure. Consistent with the results of the proliferation test, repeated EtOH exposure increased the number of apoptotic cells in the organoid. The apoptosis‐promoting effect of EtOH during retinal development has been demonstrated in rodent models [53]. Our results further confirmed the effect on human retinal development. Previous studies have shown that microphthalmia can be induced following EtOH treatment [54, 55], and the proliferation‐inhibiting and apoptosis‐promoting effects of alcohol exposure during retinal development may constitute the main underlying mechanism.

Our results confirmed that the number of PAX6^+^ human retinal cells significantly decreased following EtOH treatment. However, repeated exposures did not impede the survival of PAX6^+^ cells. A study based on Xenopus embryos showed that Pax6‐positive cells are the most vulnerable cells in both the retina and the brain [56, 57]. Alcohol concentrations as low as 0.3% could produce more than a 90% reduction in PAX6 expression [57]. Using isolated human fetal radial glial cells, another study showed that EtOH treatment significantly reduced the expression of PAX6 and proliferation of radial glial cells [58]. Our findings were consistent with these results. A recent study showed the persistent expression of PAX6 in RGCs during retinal development [59]. Therefore, we believe that the EtOH exposure‐related decrease in the number of PAX6^+^ human retinal cells was associated with the loss of RGCs.

Our results also showed that the migration of TUJ1^+^ cells within the human retinal organoid was attenuated by EtOH treatment. Using TUJ1 and BRN3A double staining, our previous study confirmed that these TUJ1^+^ cells within the inner layer of retinal organoids are RGCs. In other words, EtOH exposure impaired the migration of RGCs during development. In zebrafish, RGCs normally appear approximately 28‐h postfertilisation [60]. With EtOH treatment, RGCs did not appear at 36‐h postfertilisation [52], suggesting that EtOH exposure delayed the differentiation of RGCs. A recent study also confirmed that the retinal nerve fibre layer is thinner in young adults with FAS [12], which might be a consequence of the loss of RGCs. In addition, a study found that PAX6 could guide the intraretinal axons of RGCs and fasciculate to reach the optic disc [59]. As the aforementioned EtOH exposure‐related loss of PAX6^+^ cells was linked to RGCs, all evidence indicates that the survival, differentiation and migration of RGCs were significantly impeded by EtOH exposure during retinal development.

Following pretreatment with RSV, the proliferation‐inhibiting and apoptosis‐promoting effects of alcohol exposure were partially inhibited. The number of PAX6^+^ cells and migration of TUJ1^+^ cells also increased. Although the protective role of RSV against EtOH‐induced neural cell death has already been demonstrated in a rodent FAS model [61], our study confirmed not only the death‐preventing and growth‐promoting effects of RSV in an EtOH‐induced human retinal organoid model but also its therapeutic role in specific subtypes of retinal cells. To further explore the mechanism underlying how RSV exerts its protective effects, RNA sequencing and RT‐qPCR were performed. After enrichment analysis and subsequent check analysis, the PI3K‐AKT signalling pathway was screened. Recent robust studies have shown that RSV can play a protective role by activating the PI3K‐AKT signalling pathway, including reducing myocardial cell apoptosis [62], attenuating radiation enteritis [63], attenuating dapagliflozin‐induced renal gluconeogenesis [64] and alleviating dextran sulfate sodium‐induced acute ulcerative colitis [65]. In addition, the present study further verified the role of PI3K‐AKT signalling pathway in RSV protective effects on alcohol‐induced NRs damage. The inhibitors (LY294002) and agonists (740 Y‐P) of the PI3K‐AKT signalling pathway were used. It was demonstrated that inhibition of the PI3K‐AKT signalling pathway led to a decrease in the number of KI67‐positive cells, PAX6‐positive retinal progenitor cells and TUJ1‐positive ganglion cells. However, the agonists 740 Y‐P salvages these injuries, which exhibits comparable protective effects with RSV pretreatment. These results suggest that RSV pretreatment protects the NRs from alcohol‐induced damage by activating the PI3K‐AKT signalling pathway. Further factor‐mediating studies should be conducted to elucidate a more detailed mechanism.

Resveratrol is a polyphenol compound contained abundantly in red grape skins and has used for the treatment of metabolic diseases, such as diabetes mellitus and obesity [66]. As a natural antioxidant, RSV has been shown to promote embryonic development by inducing hormone secretion and even reduce fetal mortality in pregnant women with severe hypoxia [67, 68]. In addition, RSV also is a therapeutic strategy for preeclampsia and fetal growth restriction by increasing the velocity of uterine artery blood flow [69, 70]. The present study shows that RSV protects retina against alcohol‐induced damage by promoting proliferation and inhibiting apoptosis. For pregnant women who have a history of alcohol abuse or who inevitably drink alcohol during pregnancy, RSV supplementation may be given daily before and during pregnancy to prevent fetal alcohol retinal damage.

## Conclusion

EtOH exposure can inhibit proliferation and promote apoptosis of retinal cells in human retinal organoids. Repeated EtOH exposure further exacerbated these proliferation‐inhibiting and apoptosis‐promoting effects. Moreover, the number of PAX6^+^ cells within the inner layer of retinal organoids decreased after EtOH exposure. The migration of TUJ1^+^ cells was impeded by EtOH exposure. Pretreatment with RSV not only partially reversed the proliferation‐inhibiting and apoptosis‐promoting effects of EtOH but also increased the number of PAX6^+^ cells and improved the migration of TUJ1^+^ cells. Activation of the PI3K‐AKT signalling pathway may be the underlying mechanism for this. These results indicate that embryonic EtOH exposure can impair retinal development. Pretreatment with RSV might be an effective method for preventing alcohol‐induced damage, which may be clinically relevant for patients who are exposed to alcohol during pregnancy. Future studies should further explore the effects of alcohol on the differentiation of mature retinal cells and the therapeutic effects of RSV after alcohol exposure.

## Conflict of interest

The authors declare that the research was conducted in the absence of any commercial or financial relationships that could be construed as a potential conflict of interest.

### Peer review

The peer review history for this article is available at https://publons.com/publon/10.1002/2211‐5463.13601.

## Author contributions

LG, HL and ZY conceived and designed the study. HL and LG carried out the experiment and acquisition of data. LG and HL analysed the data and wrote the manuscript. JD, LL and WL conducted literature retrieval and screening. ZY, QZ and JX reviewed the first draft of the manuscript. JX, WY and ZL revised the manuscript and supervised the project. All authors contributed to the article and approved the final version to be submitted.

## Supporting information


**Fig. S1.** Characterisation of human embryonic stem cells (ESCs). (A–C) Brightfield images of H9‐ESCs in the feeder‐free medium on Day 1 (A), Day 3 (B) and Day 5 (C). (D–L) Immunofluorescence of H9‐ESCs by NANOG (D–F), SOX2 (G–I) and OCT4 (J–L). (M) Statistical analysis of the NANOG‐, SOX2‐ and OCT4‐positive cells. (N) Flow cytometry analysis shows the positive rates of H9‐ESCs for SSEA4 and TRA‐1‐60. (O) Alkaline phosphatase staining of H9‐ESCs. Scale bars: (A–C) 100 μm; (D–L) 20 μm; (O) 500 μm.

**Fig. S2.** Volume comparison of retinal organoids from each group at three different time points. (A–M) Representative brightfield images of organoids from each group were captured at 100× magnification on Day 30, Day 37 and Day 44. The organoids in each group became more enlarged and transparent with time (Scale bar: 200 μm). (N–P) Statistical analysis of organoid volumes in each group on Day 30, Day 37 and Day 44. No statistically significant difference in organoid volumes was found among groups at each time point by one‐way ANOVA analysis and Bonferroni's *post hoc* test (N = 15 from three experiments, *P* > 0.05). Error bars represent standard deviations. EtOH: ethanol; RSV: resveratrol.

**Fig. S3.** Apoptotic cells induced by alcohol mainly accumulated in the inner layer of the neural retina. (A–M) Representative fluorescent images of apoptotic cells indicated with TUNEL (green dot for dUTP) were taken at 1000× magnification. Nuclei were stained with DAPI (blue). Scale bar: 20 μm. EtOH: ethanol; RSV: resveratrol.

**Fig. S4.** Gene ontology (GO) enrichment analysis of the differentially expressed genes in the two groups of alcohol‐afflicted retinal organoids. (A, B) The top 10 enriched GO terms for the differentially expressed genes from RSV + EtOH1 vs. EtOH1 (A) and RSV + EtOH2 vs. EtOH2 (B) are demonstrated as bubble diagrams. The bubble size indicates the number of enriched genes, and the colour scale corresponds to the adjusted p‐value. (C, D) Network diagrams illustrate the connections between five representative GO terms and the related top genes in RSV + EtOH1 vs. EtOH1 (C) and RSV + EtOH2 vs. EtOH2 (D). Dot size corresponds to the number of genes hit by the GO term. EtOH: ethanol; RSV: resveratrol.

**Fig. S5.** Overview of differentially expressed genes between RSV + EtOH and control groups. (A, B) Volcano plots of the differentially expressed genes between RSV + EtOH1 and control groups (A) and between RSV + EtOH2 and control groups (B). Dash‐dotted lines: vertical ones represent log transformed p‐value, and horizontal ones indicated the mean expression differences of genes between RSV + EtOH 1 and control groups. Red dots are for upregulated genes and blue dots for downregulated ones. (C, D) The top 25 differentially expressed genes in RSV + EtOH1 vs Control (C) and RSV + EtOH2 vs Control (D). Colour scale bar denotes the expression levels from high (dark red) to low (dark blue). EtOH: ethanol; RSV: resveratrol.

**Fig. S6.** Enrichment analysis of differentially expressed genes between RSV + EtOH and control groups at day 44. (A, B) Bubble diagrams showed the top 10 enriched GO terms for the differentially expressed genes from RSV + EtOH1 vs. control and RSV + EtOH2 vs. control, respectively. (C, D) Bubble diagrams showed the top 10 enriched KEGG pathways for the differentially expressed genes from RSV + EtOH1 vs. control and RSV + EtOH2 vs. control, respectively. The bubble size indicates the number of genes hit in enrichment and the colour scale for the adjusted p‐value. (E, F) The enrichment plot of GSEA results for the differentially expressed genes in RSV1 + EtOH1 vs. control and RSV2 + EtOH2 vs. control, respectively. EtOH: ethanol; RSV: resveratrol.

## Data Availability

The raw data of RNA sequencing that support the findings of this study are available from the corresponding author upon reasonable request, without undue reservation.
