# Supplementary figures and images for: Protective effects of resveratrol on the ethanol‐induced disruption of retinogenesis in pluripotent stem cell‐derived organoids
Source: FEBS Open Bio. 2023 Mar 30;13(5):845–66. doi: 10.1002/2211-5463.13601 (PMC10153345; doi:10.1002/2211-5463.13601)

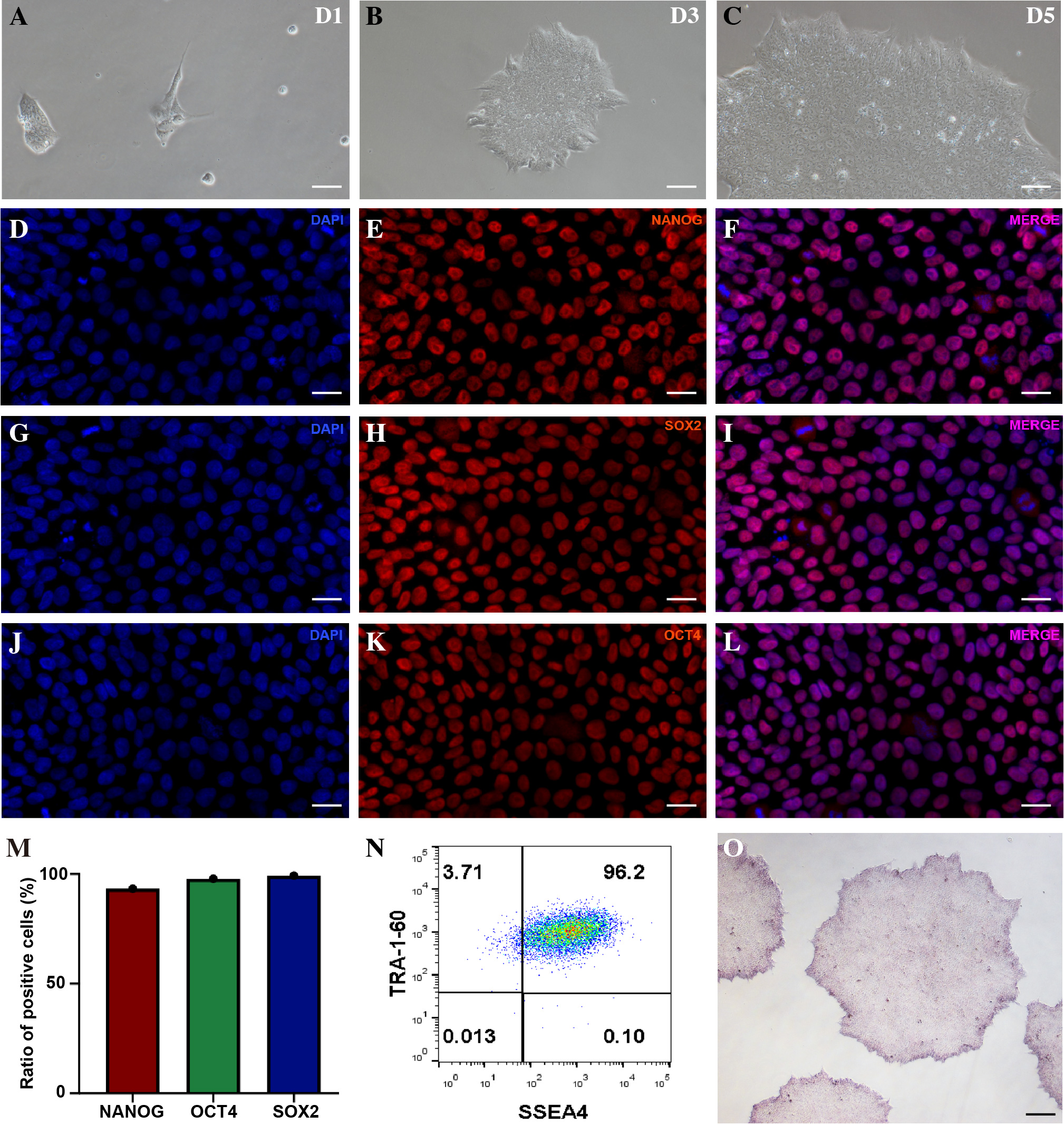

Supplement: Supplementary file 1 — Fig. S1. Characterisation of human embryonic stem cells (ESCs). (A–C) Brightfield images of H9‐ESCs in the feeder‐free medium on Day 1 (A), Day 3 (B) and Day 5 (C). (D–L) Immunofluorescence of H9‐ESCs by NANOG (D–F), SOX2 (G–I) and OCT4 (J–L). (M) Statistical analysis of the NANOG‐, SOX2‐ and OCT4‐positive cells. (N) Flow cytometry analysis shows the positive rates of H9‐ESCs for SSEA4 and TRA‐1‐60. (O) Alkaline phosphatase staining of H9‐ESCs. Scale bars: (A–C) 100 μm; (D–L) 20 μm; (O) 500 μm. [file FEB4-13-845-s005.jpg]

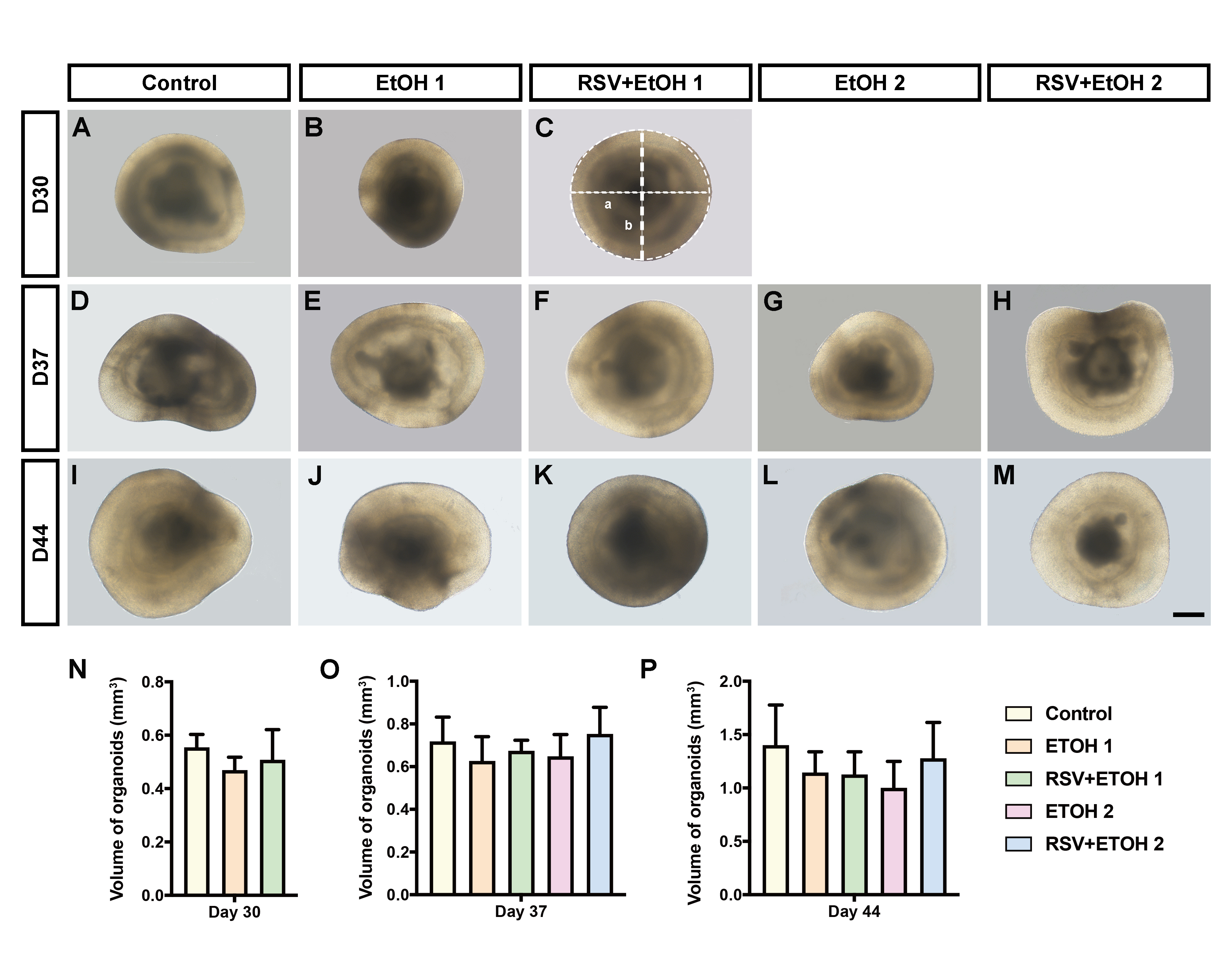

Supplement: Supplementary file 2 — Fig. S2. Volume comparison of retinal organoids from each group at three different time points. (A–M) Representative brightfield images of organoids from each group were captured at 100× magnification on Day 30, Day 37 and Day 44. The organoids in each group became more enlarged and transparent with time (Scale bar: 200 μm). (N–P) Statistical analysis of organoid volumes in each group on Day 30, Day 37 and Day 44. No statistically significant difference in organoid volumes was found among groups at each time point by one‐way ANOVA analysis and Bonferroni's post hoc test (N = 15 from three experiments, P > 0.05). Error bars represent standard deviations. EtOH: ethanol; RSV: resveratrol. [file FEB4-13-845-s001.jpg]

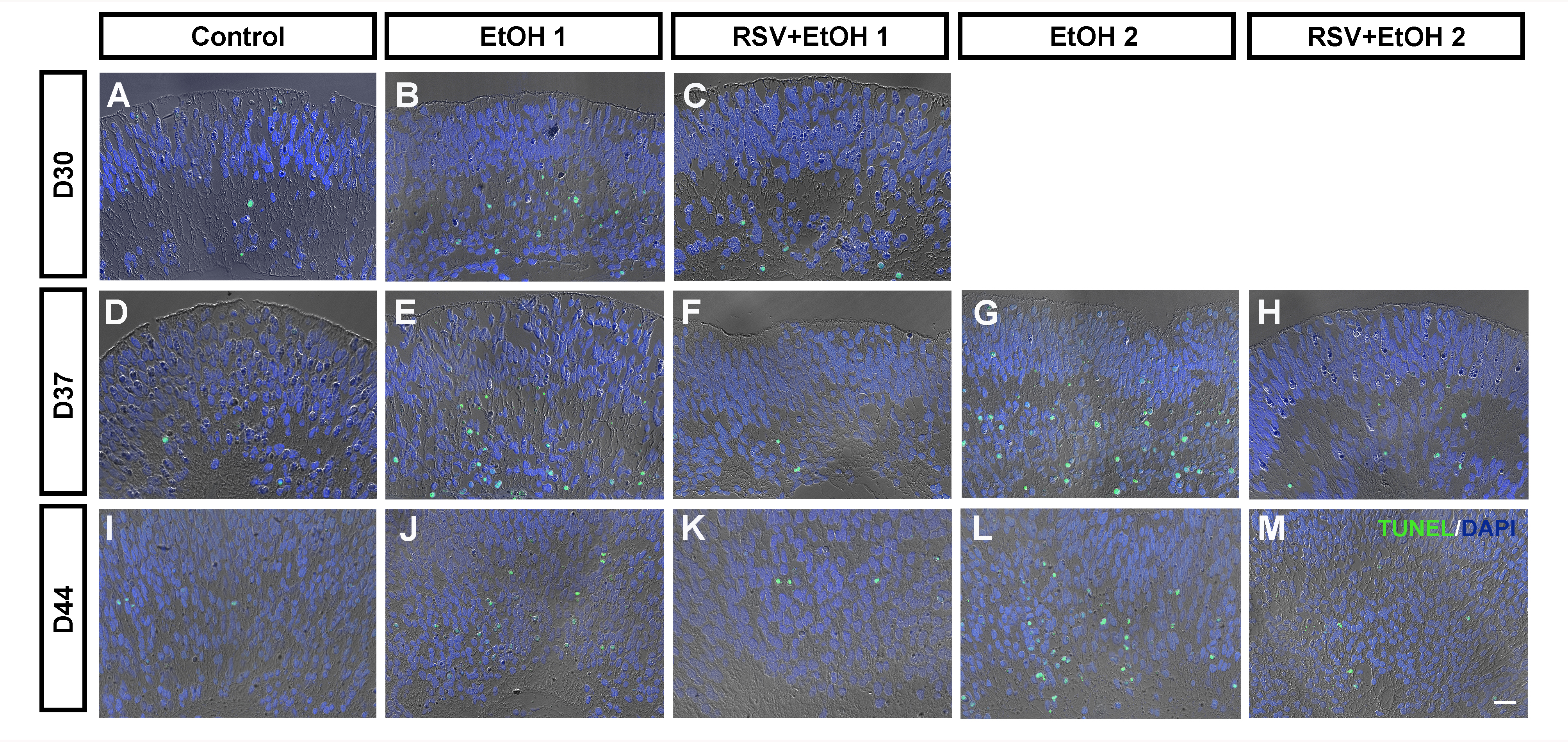

Supplement: Supplementary file 3 — Fig. S3. Apoptotic cells induced by alcohol mainly accumulated in the inner layer of the neural retina. (A–M) Representative fluorescent images of apoptotic cells indicated with TUNEL (green dot for dUTP) were taken at 1000× magnification. Nuclei were stained with DAPI (blue). Scale bar: 20 μm. EtOH: ethanol; RSV: resveratrol. [file FEB4-13-845-s003.jpg]

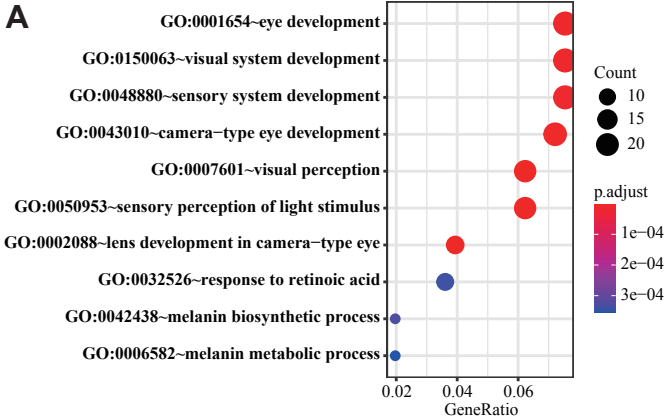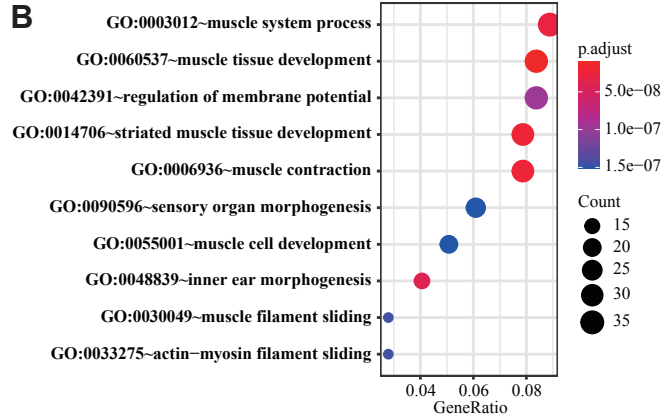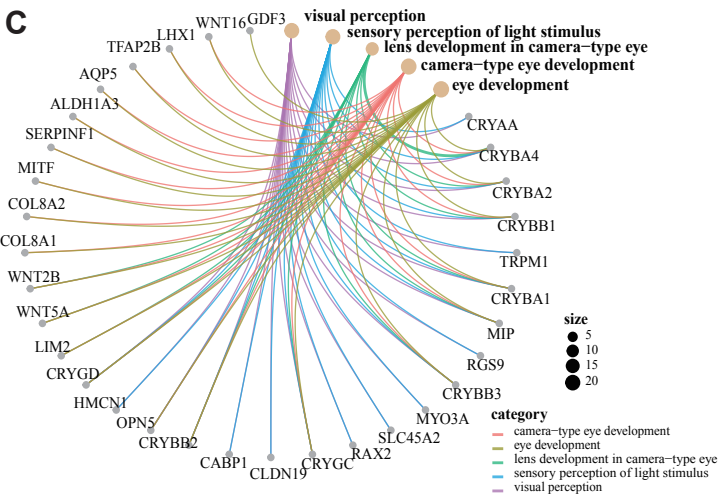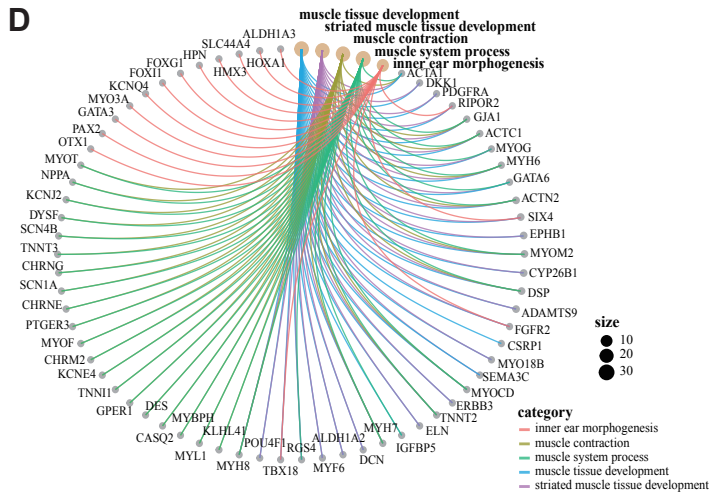

Supplement: Supplementary file 4 — Fig. S4. Gene ontology (GO) enrichment analysis of the differentially expressed genes in the two groups of alcohol‐afflicted retinal organoids. (A, B) The top 10 enriched GO terms for the differentially expressed genes from RSV + EtOH1 vs. EtOH1 (A) and RSV + EtOH2 vs. EtOH2 (B) are demonstrated as bubble diagrams. The bubble size indicates the number of enriched genes, and the colour scale corresponds to the adjusted p‐value. (C, D) Network diagrams illustrate the connections between five representative GO terms and the related top genes in RSV + EtOH1 vs. EtOH1 (C) and RSV + EtOH2 vs. EtOH2 (D). Dot size corresponds to the number of genes hit by the GO term. EtOH: ethanol; RSV: resveratrol. [file FEB4-13-845-s002.pdf]

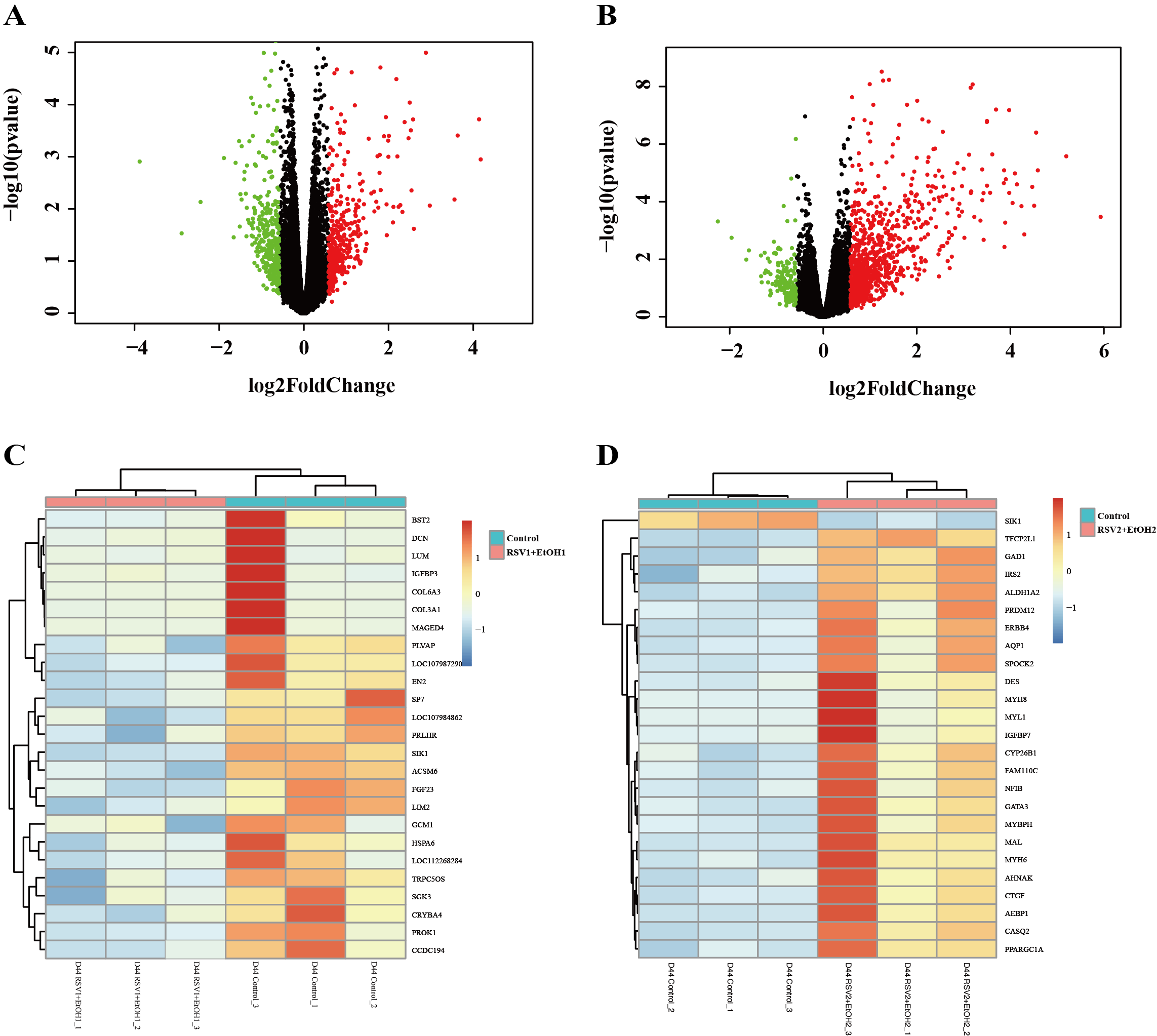

Supplement: Supplementary file 5 — Fig. S5. Overview of differentially expressed genes between RSV + EtOH and control groups. (A, B) Volcano plots of the differentially expressed genes between RSV + EtOH1 and control groups (A) and between RSV + EtOH2 and control groups (B). Dash‐dotted lines: vertical ones represent log transformed p‐value, and horizontal ones indicated the mean expression differences of genes between RSV + EtOH 1 and control groups. Red dots are for upregulated genes and blue dots for downregulated ones. (C, D) The top 25 differentially expressed genes in RSV + EtOH1 vs Control (C) and RSV + EtOH2 vs Control (D). Colour scale bar denotes the expression levels from high (dark red) to low (dark blue). EtOH: ethanol; RSV: resveratrol. [file FEB4-13-845-s004.png]

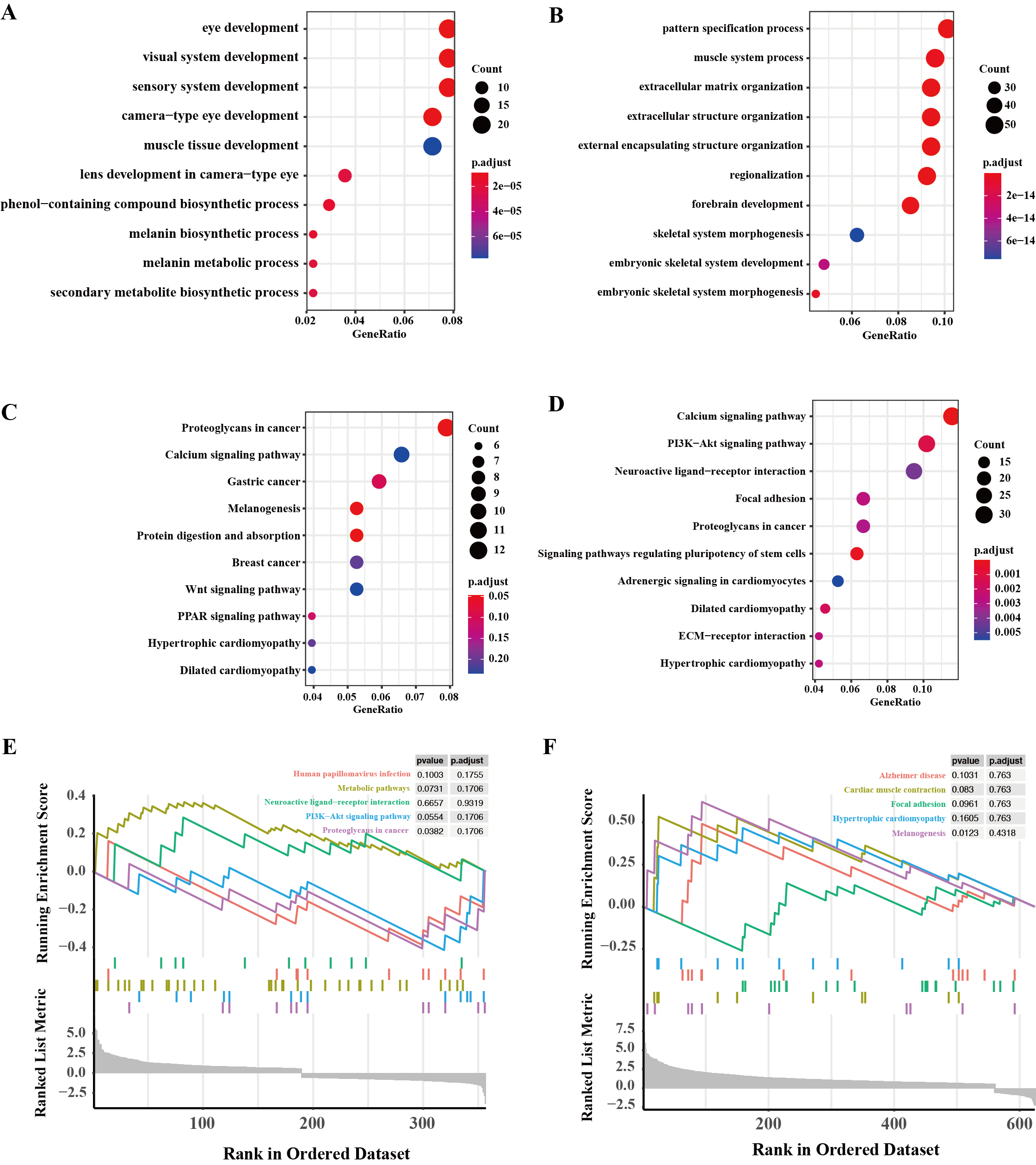

Supplement: Supplementary file 6 — Fig. S6. Enrichment analysis of differentially expressed genes between RSV + EtOH and control groups at day 44. (A, B) Bubble diagrams showed the top 10 enriched GO terms for the differentially expressed genes from RSV + EtOH1 vs. control and RSV + EtOH2 vs. control, respectively. (C, D) Bubble diagrams showed the top 10 enriched KEGG pathways for the differentially expressed genes from RSV + EtOH1 vs. control and RSV + EtOH2 vs. control, respectively. The bubble size indicates the number of genes hit in enrichment and the colour scale for the adjusted p‐value. (E, F) The enrichment plot of GSEA results for the differentially expressed genes in RSV1 + EtOH1 vs. control and RSV2 + EtOH2 vs. control, respectively. EtOH: ethanol; RSV: resveratrol. [file FEB4-13-845-s006.png]
